# Supplementary material for: Stabilization of membrane topologies by proteinaceous remorin scaffolds
Source: Nat Commun. 2023 Jan 19;14:323. doi: 10.1038/s41467-023-35976-5 (PMC9852587; doi:10.1038/s41467-023-35976-5)
Supplement: Supplementary file 1 — Supplementary Information [file 41467_2023_35976_MOESM1_ESM.pdf]

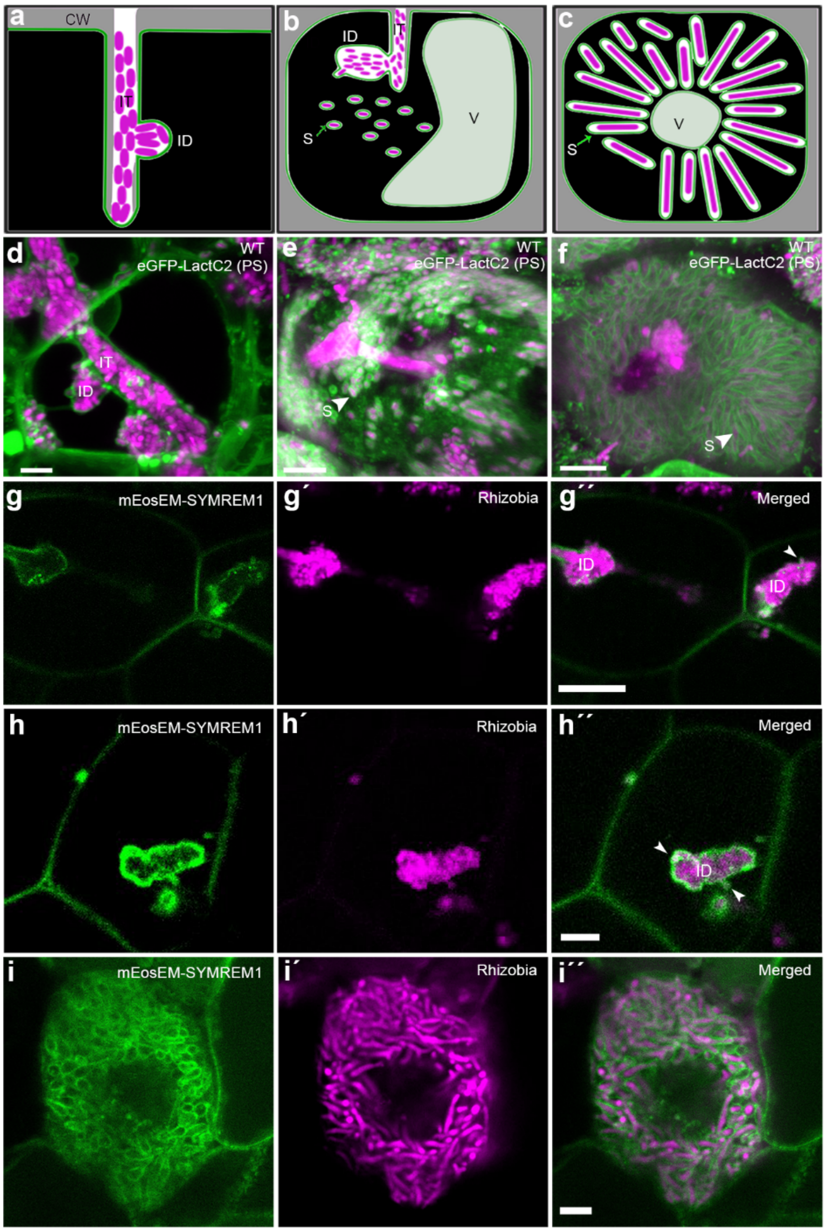

Supplementary Fig. 1. Visualization of symbiotic membranes. (a-c) Sketches of infected cells inside nodules at different stages (also used in Fig. 1). CW: cell wall; IT: infection thread; ID: infection droplet; S: symbiosome; V: vacuole. (d-f) Phosphatidylserine (PS) was labelled with a LactC2 biosensor (green) to visualize symbiotic membrane structures in wild-type (WT, A17) nodules. Rhizobia (*S. meliloti*) were expressing a mCherry marker (magenta). Arrowheads indicate symbiosomes (e, f). Images were taken as z-stacks (internal distance is 0.5 µm) and are shown as 3D projections generated by using Imaris. (g-i). Single focal plane images from a max. projection shown in Fig. 1 to visualize the localization of mEosEM-tagged SYMREM1 accumulating at bacterial droplets (g,h) and symbiosome membranes (i). The arrowheads in g´´ and h´´ indicate bacterial release sites. Scale bars indicate 5 µm. Experiments were performed on 3 independent biological replicates with at least 10 nodules checked for each replicate. The sketches (a-c) were drawn with Inkscape.

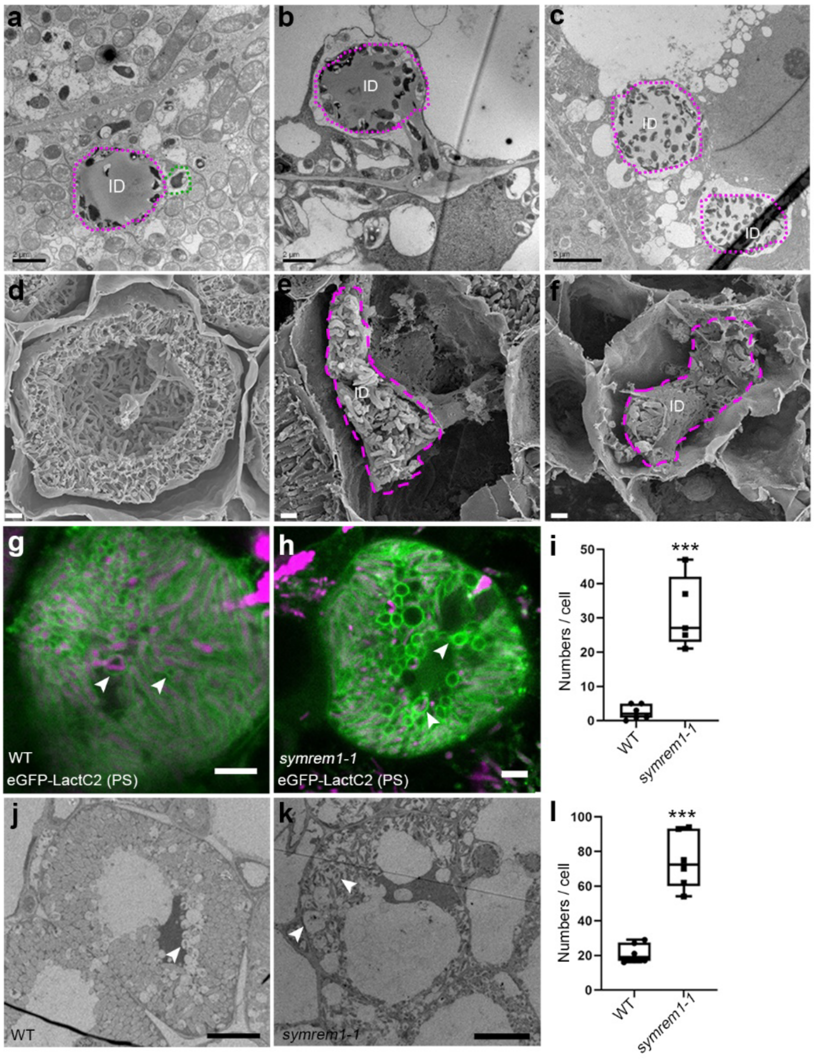

Supplementary Fig. 2. *symrem1* mutants display defects in bacterial release. (a-c) Transmission electron microscopy (TEM) showing normal rhizobial release into wild-type cells (WT, a) whereas bacteria are trapped inside the IT droplets in *symrem1* mutants (b-c). ID, infection droplet (encircled in magenta (a-c)) with bacteria being actively released (green circle (a)). (d-f) Scanning electron microscopy (SEM) showing normal rhizobial release into WT bacteroids (d) while bacteria are trapped inside the infection droplets (encircled in magenta) in *symrem1* mutants (e-f). Scale bars indicate 2 µm (a,b), 5 µm (c), 4 µm (d) and 3 µm (e,f). SEM experiments were performed on 2 biological replicates with 5 nodules being checked for each genotype and each replicate. (g-h) Visualization of symbiosome membranes using the phosphatidylserine reporter Lact-C2 (PS) in wild-type (WT) and *symrem1-1* nodules. (i) Quantification of abnormal symbiosomes (with the membrane either loosely associated with released rhizobia or without rhizobia inside, indicated by arrowheads) per cells in WT and *symrem1-1* (n = 6 for WT and n = 5 for *symrem1-1*). (j,k) TEM analysis of symbiosomes for WT (j) and *symrem1-1* (k). (l) Quantification of abnormal symbiosomes (indicated by arrowheads) based on TEM images (n = 6 for both WT and *symrem1-1*). (i,l): Box-whiskers plots with all data points shown; the boxes are extended from the 25th to 75th percentiles and the line in the middle of the box is plotted at the median. Data are means ± SE. Statistics were performed using an unpaired two-tailed t-test: ***$p < 0.001$. Scale bars indicate 5 µm in (g,h,j,k).

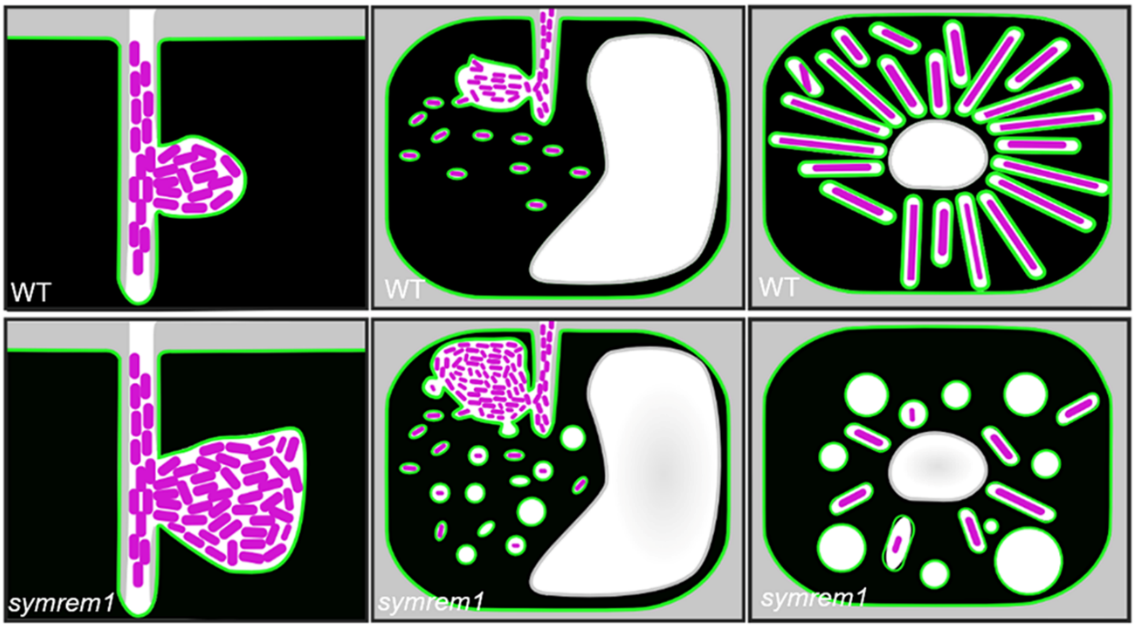

Supplementary Fig. 3. Illustration of phenotypic observations in WT vs. *symrem1* mutant nodules. Defined infection droplets from which rhizobia are released into symbiosomes with a tightly aligned symbiosome membrane are found in WT nodules. In contrast, IDs in *symrem1* mutant nodules are often less defined and symbiosome membranes are loosely assembled around the engulfed bacteria with some symbiosome membranes lacking bacteria and thus appearing as empty spheres. The sketches (a-c) were drawn with Inkscape.

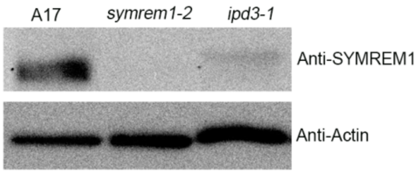

Supplementary Fig. 4. SYMREM1 protein levels are greatly reduced in *ipd3-1* mutants. Nodules from *symrem1-2* and *ipd3-1* mutant plants were harvested at 2 weeks post-inoculation with *S. meliloti* and used for total protein extraction. Western blot analysis was performed using custom-made anti-SYMREM1 peptide and anti-actin antibodies. Experiments were repeated 2 times independently with similar results.

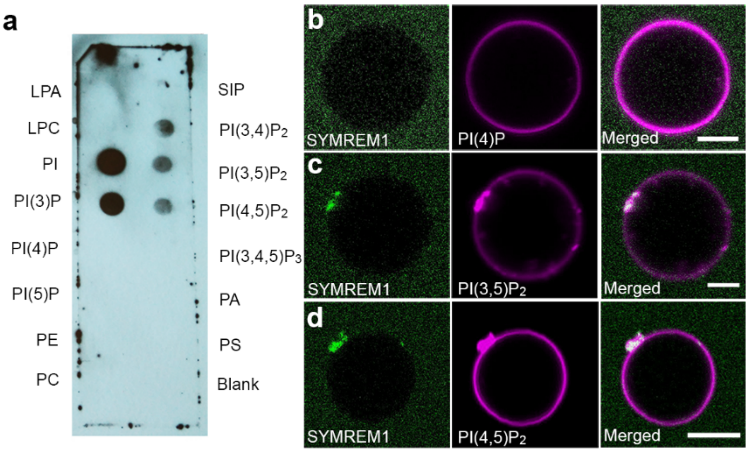

Supplementary Fig. 5. SYMREM1 binds negatively charged lipids. (a) Hybridization of recombinant His-tagged SYMREM1 to lipid-strips revealed a specific association with phosphatidylinositol (PI), phosphatidylinositol 3-phosphate PI3P and the phosphatidylinositol bisphosphates PI(3,4)P_2, PI(3,5)P_2 and PI(4,5)P_2. This assay was repeated twice with independently isolated recombinant SYMREM1 protein, of which one replicate is shown. LPA (lysophosphatidic acid); LPC (lysophosphocholine); PE (phosphatidylethanolamine); PC (phosphatidylcholine); S1P (sphingosine 1-Phosphate); PA (phosphatidic acid); PS (phosphatidylserine). (b-d) Purified His-GFP-tagged SYMREM1 protein (green) was applied to GUVs (Atto 647N-DOPE was used as membrane marker; magenta) assembled from negative charged lipids PI4P (b), PI(3,5)P_2 (c) and PI(4,5)P_2 (d). SYMREM1 bound to (c) PI(3,5)P_2- and (d) PI(4,5)P_2-comprising GUVs and induced membrane morphology changes at the binding sites. Scale bars indicate 10 µm. GUV experiments were performed twice with independently isolated SYMREM1 protein.

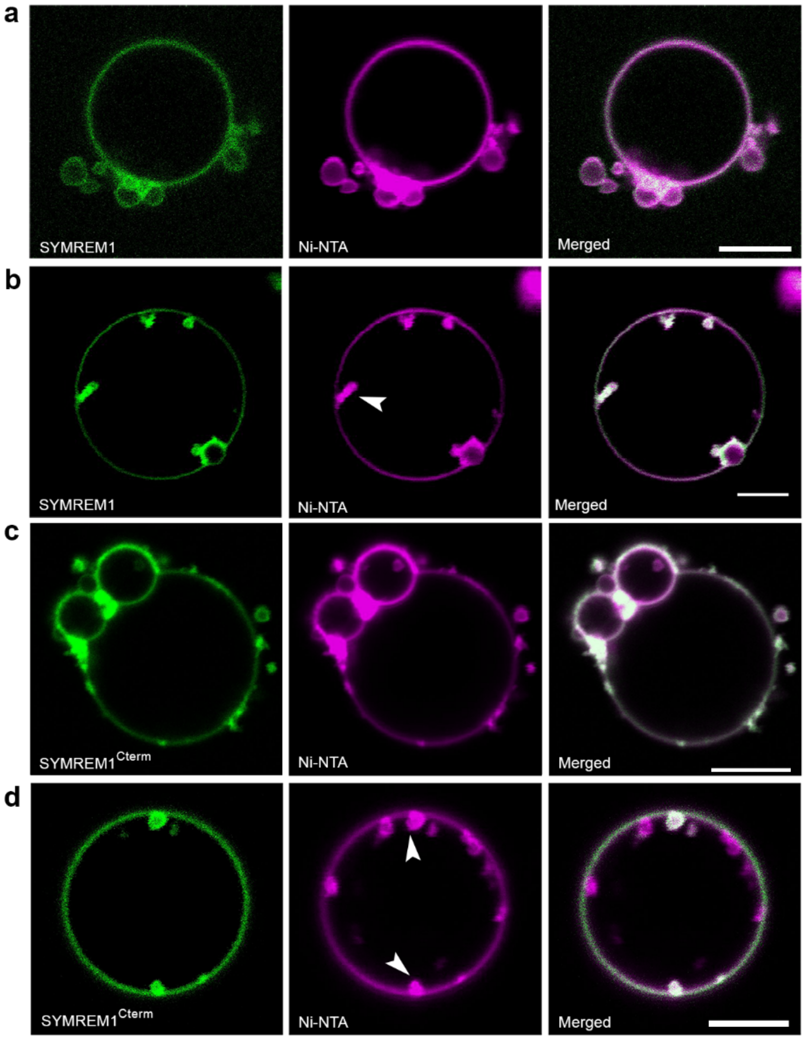

Supplementary Fig. 6. SYMREM1-induced topology changes on GUV membranes. (a-d) 18:1 DGS-NTA(Ni) (Ni-NTA) containing GUVs (Atto 647N-DOPE was used as membrane marker; magenta) were incubated with purified His-GFP-tagged SYMREM1 full-length protein (a,b) and the helical C-terminal region (SYMREM1^{Cterm}, c,d), all in green. Membrane blebs (a,c) or membrane invaginations (b,d; arrowheads) were generated on GUVs after incubation with SYMREM1 (a,b) and SYMREM1^{Cterm} (c,d). Scale bars indicate 10 µm. GUV experiments were performed twice with independently isolated SYMREM1 protein.

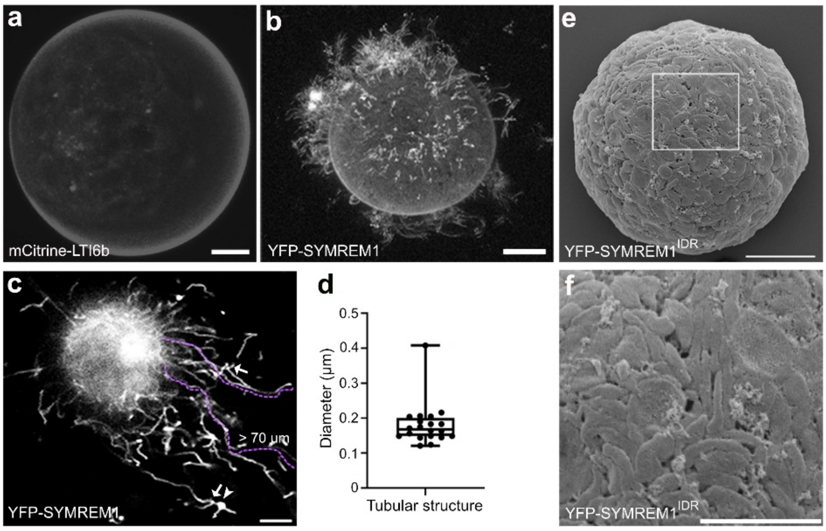

Supplementary Fig. 7. SYMREM1-induced membrane tubulation on protoplast surfaces. (a) Protoplasts expressing the transmembrane domain of LTI6b (as control) maintained a smooth surface spherical shape. (b) By contrast, strong membrane tubulation was observed on protoplasts expressing full-length SYMREM1. (c) Tube lengths (indicated by magenta lines) greatly varied with long tubes exceeding 70 µm and some tubes being branched (arrows) or swollen (arrowhead). (a-c) are maximum projections of confocal images. (d) Quantification data for tube diameters as scored on SEM images (n = 20). Box-whiskers plot with all data points shown; the box is extended from the 25th to 75th percentiles and the line in the middle of the box is plotted at the median. (e,f) SEM images showing the absence of tubes on protoplasts expressing the intrinsically disordered N-terminal region ($SYMREM1^{IDR}$), with (f) showing a close-up of the region indicated by the white box in (e). Scale bars indicate 10 µm (a,b,c,f) 20 µm (e).

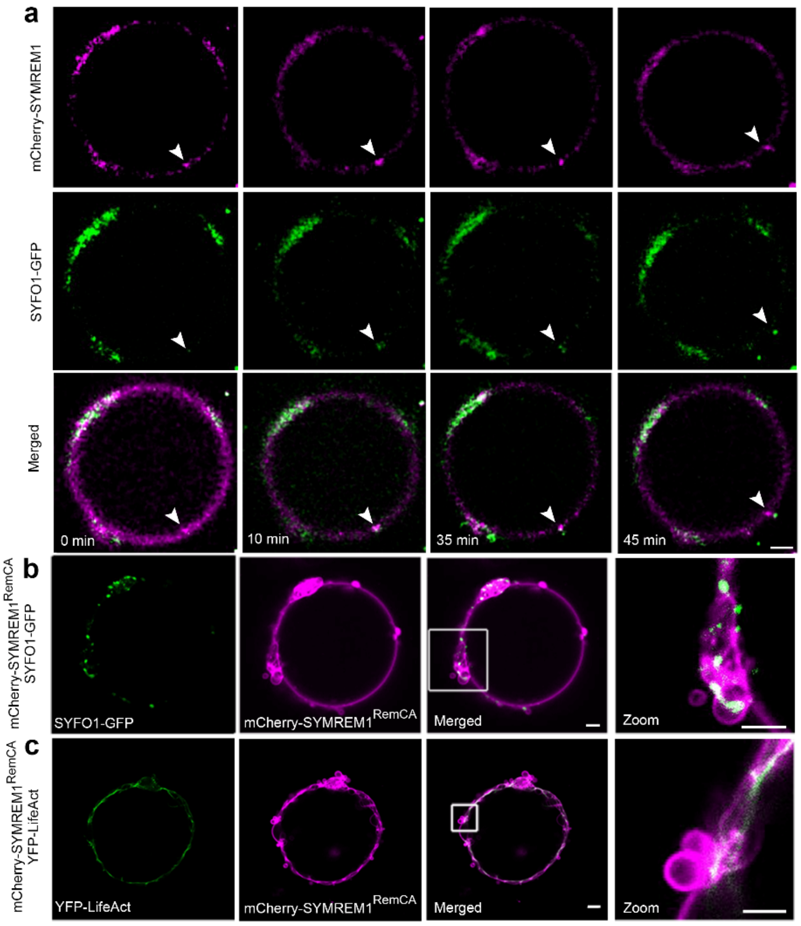

Supplementary Fig. 8. The formin SYFO1 is recruited to SYMREM1 foci over time. (a) Time-lapse confocal imaging of protoplasts obtained from *N. benthamiana* leaves co-expressing mCherry-SYMREM1 (magenta) and SYFO1-GFP (green) two days after infiltration indicated a gradual enrichment of SYFO1 in SYMREM1-positive membrane foci (arrowheads). SYFO1 (green, b) but not filamentous actin as labelled by LifeAct (green, c) was recruited into membrane blebs induced by the expression of SYMREM1^{RemCA} (magenta). Scale bars indicate 15 µm. Experiments were repeated 3 times independently with 3 protoplasts for (a) and at least 30 protoplasts for (b, c) being observed with similar results.

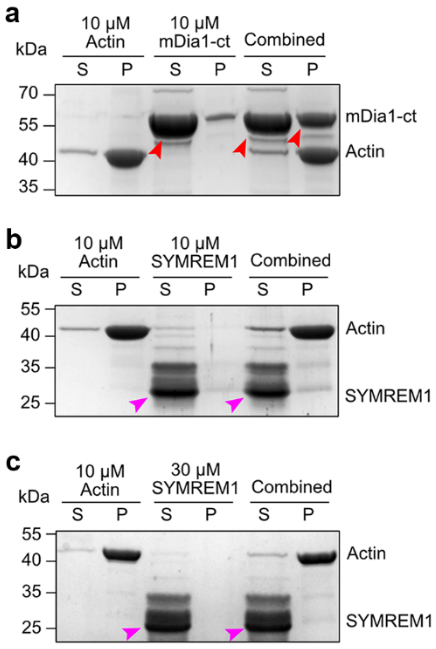

Supplementary Fig. 9. SYMREM1 and actin do not directly interact *in vitro*. Co-sedimentation assay using purified recombinant SYMREM1 and the C-terminal domain of human formin mDia1 (mDia1-ct) together with commercial G-actin. (a-c) Coomassie stained SDS gels obtained after one-hour of co-incubating G-actin in polymerization buffer with either 10 µM mDia1-ct (a; positive control), 10 µM SYMREM1 (b) and 30 µM SYMREM1 (c). Soluble (S) and pelleted (P) fractions were obtained after ultracentrifugation. Arrowheads indicate mDia1-ct (a; red) and SYMREM1 (b,c; magenta).

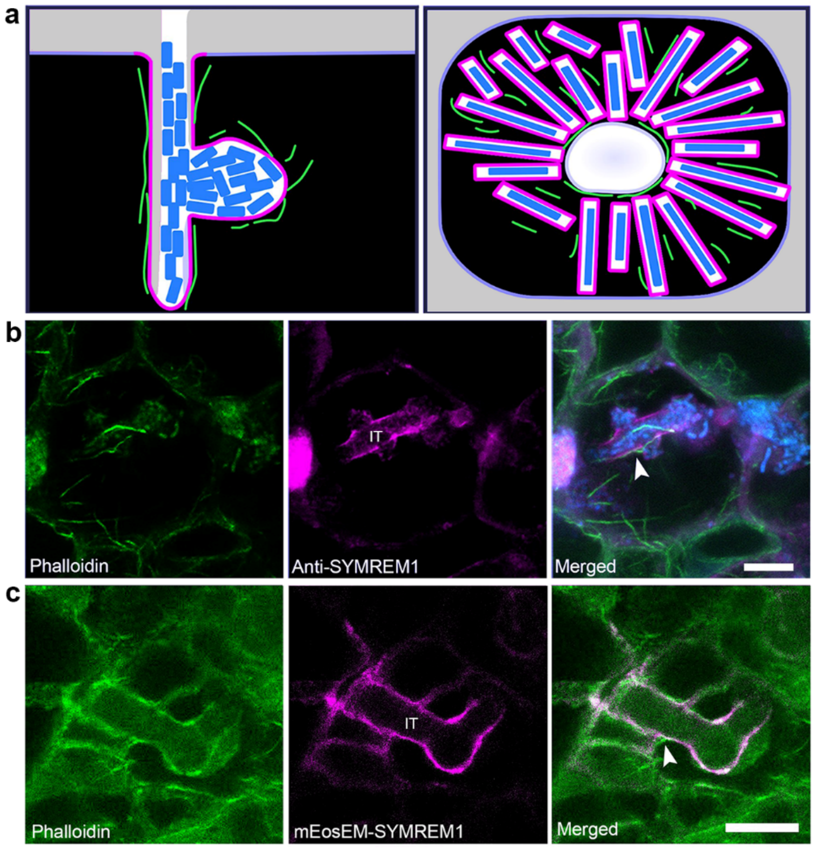

Supplementary Fig. 10. SYMREM1 and actin colocalize around infection threads inside nodules. (a) Illustrations indicate the actin pattern within infected cells at the infection zone (left) and fixation zone (right). (b) Cells within the infection zone of two weeks old *M. truncatula* WT nodules were stained with Alexa Fluor™ 488 Phalloidin (green) and immunolabelled with a SYMREM1 antibody (magenta). (c) Cells of the same zone and nodule age but ectopically expressing an mEOSEM-SYMREM1 fusion protein (magenta) and counterstained with Alexa Fluor™ 568 Phalloidin (green). Arrowheads (in b and c) indicate where the actin filaments are associated with SYMREM1. Scale bars indicate 5 µm. Experiments were performed on two biological replicates, both yielding similar results. Images were taken as z-stacks (internal distance is 0.5 µm) and are shown as maximum projections. IT: infection thread. The sketches (in a) were drawn with Inkscape.

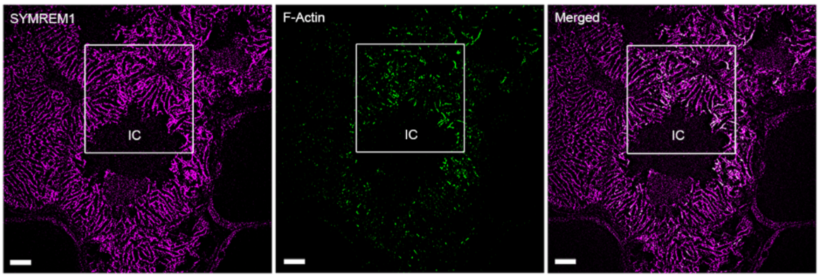

Supplementary Fig. 11. Raw images from structured illumination microscopy (SIM) showing the symbiosome membrane structures in fully colonized cells. The region marked with a white box was used for super-resolution analysis in Fig. 6f. IC: infected cell. Scale bars indicate 5 μm.

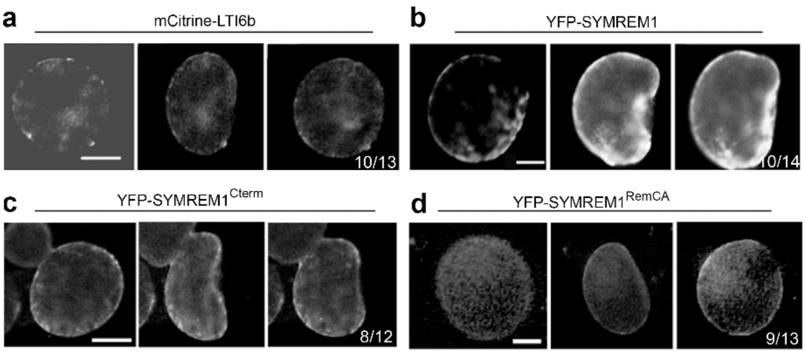

Supplementary Fig. 12. SYMREM1 stabilizes negatively curved membrane indentations. Wall-less protoplasts expressing the membrane marker mCitrine-LTI6b (a; control) and YFP-SYMREM1^{RemCA} (d) re-inflated immediately after 30 minutes of micro-capillary-based indentation, while the great majority of those expressing YFP-SYMREM1 (b) and YFP-SYMREM1^{Cterm} (c) retained the induced membrane curvature. Scale bars indicate 25 μm. Data were collected based on 4 biological replicates. Numbers indicate frequencies of observed patterns.

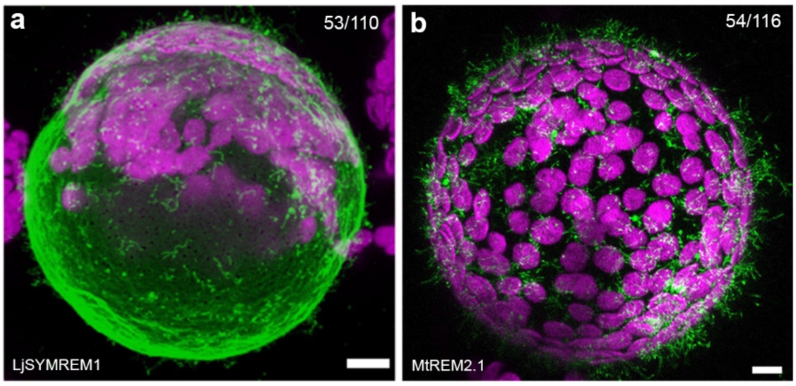

Supplementary Fig. 13. LjSYMREM1 and MtREM2.1 efficiently induce membrane tubes in protoplasts. (a-b) *N. benthamiana* leaf protoplasts ectopically expressing YFP-LjSYMREM1 (a) and GFP-MtREM2.1 (b) developed multiple membrane tubes. Magenta signals derive from chlorophyll autofluorescence within chloroplasts. Images were taken as z-stacks (internal distance is 0.5 μm) and are shown as maximum projections. Scale bars indicate 5 μm. Data were collected based on 3 biological replicates. Numbers indicate frequencies of observed patterns.

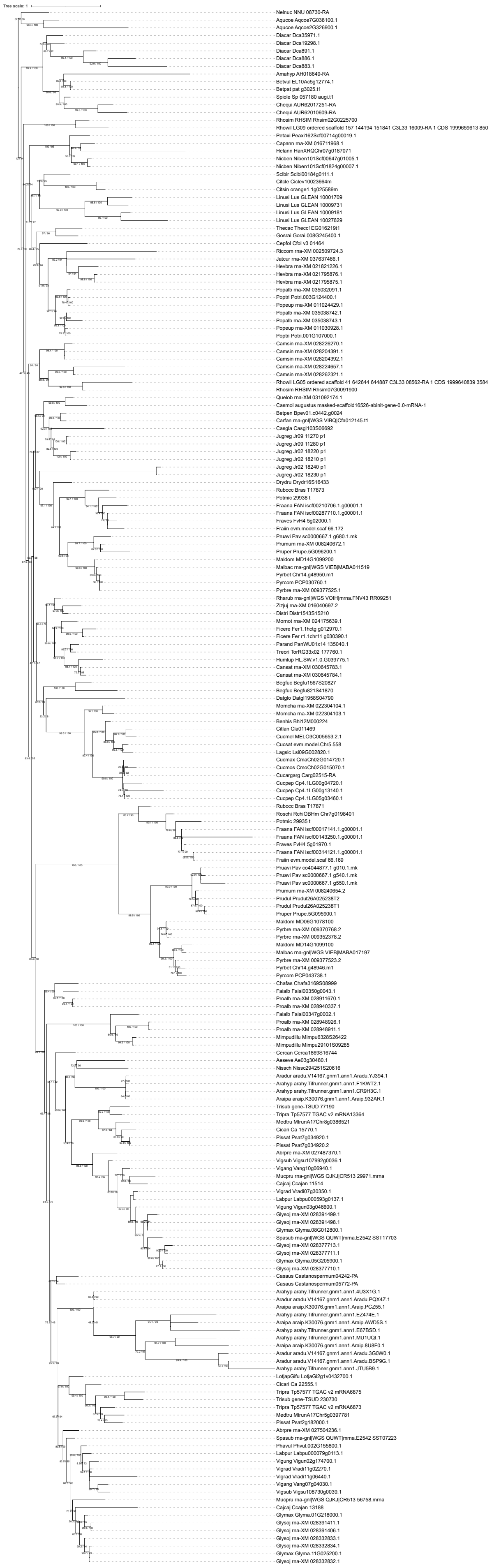

Tree scale: 1
Nelnuc NNU 08730-RA
Aqucoe Aqcoe7G038100.1
Aqucoe Aqcoe2G326900.1
Diacar Dca35971.1
Diacar Dca19298.1
Diacar Dca891.1
Diacar Dca886.1
Diacar Dca883.1
Amahyp AH018649-RA
Betvul EL10Ac5g12774.1
Betpat pat g3025.t1
Spiole Sp 057180 augi.t1
Chequi AUR62017251-RA
Chequi AUR62010609-RA
Rhosim RHSIM Rhsim02G0225700
Rhowil LG09 ordered scaffold 157 144194 151841 C3L33 16009-RA 1 CDS 1999659613 850
Petaxi Peaxi162Scf00714g00019.1
Capann rna-XM 016711968.1
Helann HanXRQChr07g0187071
Nicben Niben101Scf00647g01005.1
Nicben Niben101Scf01824g00007.1
Sclbir Sclbi00184g0111.1
Citcle Ciclev10023664m
Citsin orange1.1g025589m
Linusi Lus GLEAN 10001709
Linusi Lus GLEAN 10009731
Linusi Lus GLEAN 10009181
Linusi Lus GLEAN 10027629
Thecac Thecc1EG016219t1
Gosrai Gorai.008G245400.1
Cepfol Cfol v3 01464
Riccom rna-XM 002509724.3
Jatcur rna-XM 037637466.1
Hevbra rna-XM 021821226.1
Hevbra rna-XM 021795876.1
Hevbra rna-XM 021795875.1
Popalb rna-XM 035032091.1
Poptri Potri.003G124400.1
Popeup rna-XM 011024429.1
Popalb rna-XM 035038742.1
Popalb rna-XM 035038743.1
Popeup rna-XM 011030928.1
Poptri Potri.001G107000.1
Camsin rna-XM 028226270.1
Camsin rna-XM 028204391.1
Camsin rna-XM 028204392.1
Camsin rna-XM 028224657.1
Camsin rna-XM 028262321.1
Rhowil LG05 ordered scaffold 41 642644 644887 C3L33 08562-RA 1 CDS 1999640839 3584
Rhosim RHSIM Rhsim07G0091900
Quelob rna-XM 031092174.1
Casmol augustus masked-scaffold16526-abinit-gene-0.0-mRNA-1
Betpen Bpev01.c0442.g0024
Carfan rna-gnl|WGS VIBQ|Cfa012145.t1
Casgla Casgl103S06692
Jugreg Jr09 11270 p1
Jugreg Jr09 11280 p1
Jugreg Jr02 18220 p1
Jugreg Jr02 18210 p1
Jugreg Jr02 18240 p1
Jugreg Jr02 18230 p1
Drydru Drydr16S16433
Rubocc Bras T17873
Potmic 29938 t
Fraana FAN iscf00210706.1.g00001.1
Fraana FAN iscf00287710.1.g00001.1
Fraves FvH4 5g02000.1
Fraiin evm.model.scaf 66.172
Pruavi Pav sc0000667.1 g680.1.mk
Prumum rna-XM 008240672.1
Pruper Prupe.5G096200.1
Maldom MD14G1099200
Malbac rna-gnl|WGS VIEB|MABA011519
Pyrbet Chr14.g48950.m1
Pyrcom PCP030760.1
Pyrbre rna-XM 009377525.1
Rharub rna-gnl|WGS VOIH|mrna.FNV43 RR09251
Zizjuj rna-XM 016040697.2
Distri Distr1543S15210
Mornot rna-XM 024175639.1
Ficere Fer1.1hctg g012970.1
Ficere Fer r1.chr11 g030390.1
Parand PanWU01x14 135040.1
Treori TorRG33x02 177760.1
Humlup HL.SW.v1.0.G039775.1
Cansat rna-XM 030645783.1
Cansat rna-XM 030645784.1
Begfuc Begfu1567S20827
Begfuc Begfu821S41870
Datglo Datgl1958S04790
Momcha rna-XM 022304104.1
Momcha rna-XM 022304103.1
Benhis Bhi12M000224
Citlan Cla011469
Cucmel MELO3C005653.2.1
Cucsat evm.model.Chr5.558
Lagsic Lsi09G002820.1
Cucmax CmaCh02G014720.1
Cucmos CmoCh02G015070.1
Cucargarg Carg02515-RA
Cucpep Cp4.1LG00g04720.1
Cucpep Cp4.1LG00g13140.1
Cucpep Cp4.1LG05g03460.1
Rubocc Bras T17871
Roschi RchiOBHm Chr7g0198401
Potmic 29935 t
Fraana FAN iscf00017141.1.g00001.1
Fraana FAN iscf00143250.1.g00001.1
Fraves FvH4 5g01970.1
Fraana FAN iscf00314121.1.g00001.1
Fraiin evm.model.scaf 66.169
Pruavi Pav co4044877.1 g010.1.mk
Pruavi Pav sc0000667.1 g540.1.mk
Pruavi Pav sc0000667.1 g550.1.mk
Prumum rna-XM 008240654.2
Prudul Prudul26A025238T2
Prudul Prudul26A025238T1
Pruper Prupe.5G095900.1
Maldom MD06G1078100
Pyrbre rna-XM 009370768.2
Pyrbre rna-XM 009352378.2
Maldom MD14G1099100
Malbac rna-gnl|WGS VIEB|MABA017197
Pyrbre rna-XM 009377523.2
Pyrbet Chr14.g48946.m1
Pyrcom PCP043738.1
Chafas Chafa3169S08999
Faialb Faial00350g0043.1
Proalb rna-XM 028911670.1
Proalb rna-XM 028940337.1
Faialb Faial00347g0002.1
Proalb rna-XM 028948926.1
Proalb rna-XM 028948911.1
Mimpudillu Mimpu6328S26422
Mimpudillu Mimpu29101S09285
Cercan Cerca1869S16744
Aeseve Ae03g30480.1
Nissch Nissc29425IS20616
Aradur aradu.V14167.gnm1.ann1.Aradu.YJ394.1
Arahyp arahy.Tifrunner.gnm1.ann1.F1KWT2.1
Arahyp arahy.Tifrunner.gnm1.ann1.CR9H3C.1
Araipa araip.K30076.gnm1.ann1.Araip.932AR.1
Trisub gene-TSUD 77190
Tripra Tp57577 TGAC v2 mRNA13364
Medtru MtrunA17Chr8g0386521
Cicari Ca 15770.1
Pissat Psat7g034920.1
Pissat Psat7g034920.2
Abrpre rna-XM 027487370.1
Vigsub Vigsu107992g0036.1
Vigang Vang10g06940.1
Mucpru rna-gnl|WGS QJKJ|CR513 29971.mrna
Cajcaj Ccajan 11514
Vigrad Vradi07g30350.1
Labpur Labpu000593g0137.1
Vigung Vigun03g046600.1
Glysoj rna-XM 028391499.1
Glysoj rna-XM 028391498.1
Glymax Glyma.08G012800.1
Spasub rna-gnl|WGS QUWT|mrna.E2542 SST17703
Glysoj rna-XM 028377713.1
Glysoj rna-XM 028377711.1
Glymax Glyma.05G205900.1
Glysoj rna-XM 028377710.1
Casaus Castanospermum04242-PA
Casaus Castanospermum05772-PA
Arahyp arahy.Tifrunner.gnm1.ann1.4U3X1G.1
Aradur aradu.V14167.gnm1.ann1.Aradu.PQX4Z.1
Araipa araip.K30076.gnm1.ann1.Araip.PCZ55.1
Arahyp arahy.Tifrunner.gnm1.ann1.EZ474E.1
Araipa araip.K30076.gnm1.ann1.Araip.AWD5S.1
Arahyp arahy.Tifrunner.gnm1.ann1.E67BSD.1
Arahyp arahy.Tifrunner.gnm1.ann1.MU1UQI.1
Araipa araip.K30076.gnm1.ann1.Araip.8U8F0.1
Aradur aradu.V14167.gnm1.ann1.Aradu.3G0W0.1
Aradur aradu.V14167.gnm1.ann1.Aradu.BSP9G.1
Arahyp arahy.Tifrunner.gnm1.ann1.JTU5B9.1
LotjapGifu LotjaGi2g1v0432700.1
Cicari Ca 22555.1
Tripra Tp57577 TGAC v2 mRNA6875
Trisub gene-TSUD 230730
Tripra Tp57577 TGAC v2 mRNA6873
Medtru MtrunA17Chr5g0397781
Pissat Psat2g182000.1
Abrpre rna-XM 027504236.1
Spasub rna-gnl|WGS QUWT|mrna.E2542 SST07223
Phavul Phvul.002G155800.1
Labpur Labpu000079g0113.1
Vigung Vigun02g174700.1
Vigrad Vradi11g02270.1
Vigrad Vradi11g06440.1
Vigang Vang07g04030.1
Vigsub Vigsu108730g0039.1
Mucpru rna-gnl|WGS QJKJ|CR513 56758.mrna
Cajcaj Ccajan 13188
Glymax Glyma.01G218000.1
Glysoj rna-XM 028391411.1
Glysoj rna-XM 028391406.1
Glysoj rna-XM 028332833.1
Glysoj rna-XM 028332834.1
Glymax Glyma.11G025200.1
Glysoj rna-XM 028332832.1

Supplementary Fig. 14. Maximum likelihood tree of SYMREM1 and (co-)orthologs in Eudicots (model: VT+R7; log-likelihood: -33187.1885). The tree is rooted on basal Eudicot species. Branches of direct orthologs of SYMREM1, deriving from the Papilionoideae duplication, are highlighted in blue. Branches supports are indicated as follow: sh-aLRT/UltraFastBootstraps. Species names are indicated by a six-letter prefix (see also Supplementary Table 2).

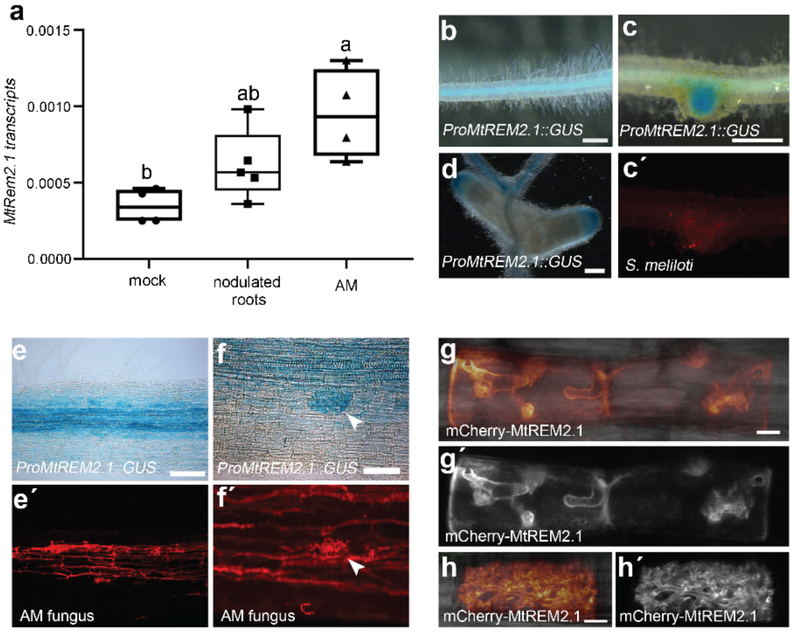

Supplementary Fig. 15. Expression and localization analysis of MtREM2.1. (a) *MtREM2.1* transcripts were determined in whole roots (wild-type R108) 5 weeks after mock treatment (mock; n=4) and 7 dpi with *S. meliloti* CFP (nodulated roots; n=5) or 5 weeks inoculated with the AM fungus *R. irregularis* (AM; n=4). Box-whiskers plot with all data points shown; the boxes are extended from the 25th to 75th percentiles and the line in the middle of the box is plotted at the median. For statistical analysis, a one-way ANOVA test was performed followed by a TukeyHSD (significant differences indicated with small letters). (b-f) Analysis of *MtREM2.1* promoter activation using a *ProMtREM2.1*::GUS reporter in transformed roots of the R108 wild-type background. (b) Weak GUS staining was detected in the vascular tissue under mock conditions. Upon inoculation with *S. meliloti*, GUS activity was found in uninfected nodule primordia 7-10 dpi (c and c´) and was later restricted to the meristematic and distal zone II of 28 days old mature nodules (d). (e,f) At 4-5 wpi with *R. irregularis*, fully colonized roots showed GUS activity in cortical cells and increased staining in arbuscule-containing cells (e and e´, f and f´. Fungal structures were stained with WGA-Alexa594 (e´-f´); arrowhead (in f and f´) indicates an arbuscule-containing cell. (g,h) Localization pattern of mCherry-MtREM2.1 expressed under the control of the native *ProMtRem2.1* promoter in stable transgenic *M. truncatula* plants (wild-type R108) at 2 wpi with *R. irregularis*. MtREM2.1 specifically labelled the symbiotic membrane surrounding intracellular hyphae with one or few branches (g and g´) as well as extensively branched arbuscules (g and g´). (g, h) are maximum projections of confocal images, (g and h) are overlays of the mCherry channel (false colored with LUT Glow) and bright field, (g´and h´) show only the mCherry channel (false colored with LUT Grays). Scale bars=500µm (b,c,d), 100µm (e,f) and 10µm (g,h).
