# Supplementary material for: Stabilization of membrane topologies by proteinaceous remorin scaffolds
Source: Nat Commun. 2023 Jan 19;14:323. doi: 10.1038/s41467-023-35976-5 (PMC9852587; doi:10.1038/s41467-023-35976-5)
Supplement: Supplementary file 3 — Description of Additional Supplementary Files [file 41467_2023_35976_MOESM3_ESM.pdf]

Description of Additional Supplementary Files:

Supplementary Data 1: Results of the branch model analysis for signature of selection acting on the SYMREM1 Caryphyllales clade. Analyzed sequences and sites indicated the number of sequences and sites analyzed corresponding to the species/tree selected. The tested branches column indicates which clade was targeted as 'Foreground' to compare selective pressure against the rest of the tree. K represent the selection intensity parameter where a significant result of $k>1$ indicates that selection strength has been intensified along the test branches, and a significant result of $k<1$ indicates that selection strength has been relaxed along the test branches. LRT represent the Likelihood Ratio Test to comparing the alternative and null models with the corresponding p-value.

Supplementary Data 2: List of species and origin of sequences used for the phylogenetic analysis. AMS= arbuscular mycorrhiza symbiosis, RNS= root nodule symbiosis, OM= Orchid mycorrhiza, EcM= ectomycorrhiza, IT= infection threads, NFN= nitrogen-fixing nodule.

Supplementary Data 3: Genetic constructs created and used within this study and corresponding gene IDs. ProUbi= Lotus Ubiquitin10 promoter.

Supplementary Data 4: Primers used in the study.
