# Supplementary material for: Stabilization of membrane topologies by proteinaceous remorin scaffolds
Source: Nat Commun. 2023 Jan 19;14:323. doi: 10.1038/s41467-023-35976-5 (PMC9852587; doi:10.1038/s41467-023-35976-5)
Supplement: Supplementary file 5 — Supplementary Data 2 [file 41467_2023_35976_MOESM5_ESM.pdf]

Supplementary Table 2. List of species and origin of sequences used for the phylogenetic analysis. AMS= arbuscular mycorrhiza symbiosis, RNS= root nodule symbiosis, OM= Orchid mycorrhiza, EcM= ectomycorrhiza, IT= infection threads, NFN= nitrogen-fixing nodule

cds_prot_db	abbr	species_abbreviation	lineage	Species name	Group	Order	Family	Angio lineage	Source	AMS	RNS	InfectionThr	OM	Ericoid/Ericoid-like	Extracellular symbiosis	Cyanobacteria	EcM	Endosymbiosis	[illegible]	embargo	genome_version
	Abrpre	abrpre	angiosperm	Abrus precatorius	Angiosperms	Fabales	Fabaceae	NFN	0	1	1	IT	0	0	0	0	0	1	0	0	
	Alnglu	alnglu	angiosperm	Alnus glutinosa	Angiosperms	Fagales	Betulaceae	NFN	10.1126/science.aat1743	1	1	IT	0	0	1	0	1	1	0	0	
	Amahyp	amahyp	angiosperm	Amaranthus hypochondriacus	Angiosperms	Caryophyllales	Amaranthaceae	Eudicots non NFN	10.3835/plantgenome2015.07.0062	0	0	never_IT	0	0	0	0	0	0	1	0	
	Ambtri	ambtri	angiosperm	Amborella trichopoda	Angiosperms	Amborellales	Amborellaceae	BasalAngiosperms	10.1126/science.1241089	1	0	never_IT	0	0	0	0	0	1	0	0	
	Anacom	anacom	angiosperm	Ananas comosus	Angiosperms	Poales	Bromeliaceae	Monocots	10.1038/ng.3435	1	0	never_IT	0	0	0	0	0	1	0	0	
	AntagrBONN	antagrBONN	bryophytes	Anthoceros agrestis	Hornworts	Anthocerotales	Anthocerataceae	0	10.1038/s41477-020-0618-2	1	0	never_IT	0	0	0	0	0	1	0	0	
	AntagrOXF	antagrOXF	bryophytes	Anthoceros agrestis	Hornworts	Anthocerotales	Anthocerataceae	0	10.1038/s41477-020-0618-2	1	0	never_IT	0	0	0	0	0	1	0	0	
	Antpun	antpun	bryophytes	Anthoceros punctatus	Hornworts	Anthocerotales	Anthocerataceae	0	10.1038/s41477-020-0618-2	1	0	never_IT	0	0	0	0	0	1	0	0	
	Aposhe	aposhe	angiosperm	Apostasia shenzhenica	Angiosperms	Asparagales	Orchidaceae	Monocots	10.1038/nature23897	0	0	never_IT	1	0	0	0	0	1	0	0	
	Aqucoe	aqucoe	angiosperm	Aquilegia coerulea	Angiosperms	Ranunculales	Ranunculaceae	BasalEudicots	10.7554/eLife.36426	1	0	never_IT	0	0	0	0	0	1	1	0	
	Aradur	aradur	angiosperm	Arachis duranensis	Angiosperms	Fabales	Fabaceae	NFN	10.1038/ng.3517	1	1	no_IT	0	0	0	0	0	1	0	0	
	Arahal	arahal	angiosperm	Arabidopsis halleri	Angiosperms	Brassicales	Brassicaceae	Eudicots non NFN	10.5061/dryad.gn4hh	0	0	never_IT	0	0	0	0	0	0	1	0	
	Arahyp	arahyp	angiosperm	Arachis hypogaea	Angiosperms	Fabales	Fabaceae	NFN	10.25739/hb5x-wx74	1	1	no_IT	0	0	0	0	0	1	0	0	
	Araipa	araipa	angiosperm	Arachis ipaensis	Angiosperms	Fabales	Fabaceae	NFN	10.1038/ng.3517	1	1	no_IT	0	0	0	0	0	1	0	0	
	Aralyr	aralyr	angiosperm	Arabidopsis lyrata	Angiosperms	Brassicales	Brassicaceae	Eudicots non NFN	10.1038/ng.807	0	0	never_IT	0	0	0	0	0	0	1	0	
	Aratha	aratha	angiosperm	Arabidopsis thaliana	Angiosperms	Brassicales	Brassicaceae	Eudicots non NFN	10.1093/nar/gkr1090	0	0	never_IT	0	0	0	0	0	0	1	0	
	Auxpro	auxpro	chlorophyta	Auxenochlorella protothecoides	Chlorophyta	Chlorellales	Chlorellaceae	0	10.1186/1471-2164-15-582	0	0	never_IT	0	0	0	0	0	0	1	0	
	Azofil	azofil	ferns	Azolla filiculoides	Leptosporangiate	Salviniales	Salviniaceae	0	10.1038/s41477-018-0188-8	0	0	never_IT	0	0	1	1	0	0	0	0	
	Batpra	batpra	chlorophyta	Bathycoccus prasinos RCC1105	Chlorophyta	Mamiellales	Bathycoccaceae	0	[illegible]	0	0	never_IT	0	0	0	0	0	0	1	0	
	Begfuc	begfuc	angiosperm	Begonia fuchsioides	Angiosperms	Cucurbitales	Begoniaceae	NFN	10.1126/science.aat1743	1	0	no_IT	0	0	0	0	0	1	0	0	
	Benhis	benhis	angiosperm	Benincasa hispida	Angiosperms	Cucurbitales	Cucurbitaceae	NFN	10.1038/s41467-019-13185-3	1	0	no_IT	0	0	0	0	0	1	0	0	
	Betpat	betpat	angiosperm	Beta patula	Angiosperms	Caryophyllales	Amaranthaceae	0	10.1111/tpj.14413	0	0	never_IT	0	0	0	0	0	0	1	0	
	Betpen	betpen	angiosperm	Betula pendula	Angiosperms	Fagales	Fagaceae	NFN	0	0	0	no_IT	0	0	1	0	1	0	0	0	
	Betvul	betvul	angiosperm	Beta vulgaris ssp vulgaris KWS2320	Angiosperms	Caryophyllales	Amaranthaceae	Eudicots non NFN	10.1101/2020.09.15.298315	0	0	never_IT	0	0	0	0	0	0	1	0	
	Boestr	boestr	angiosperm	Boechera stricta	Angiosperms	Brassicales	Brassicaceae	Eudicots non NFN	Unpublished - Phytozome	0	0	never_IT	0	0	0	0	0	0	1	1	1,2
	Botbra	botbra	chlorophyta	Botryococcus braunii	Chlorophyta	Trebouxiales	Botryococcaceae	0	Unpublished - Phytozome	0	0	never_IT	0	0	0	0	0	0	1	1	2,1
	Bradis	bradis	angiosperm	Brachypodium distachyon	Angiosperms	Poales	Poaceae	Monocots	10.1038/nature08747	1	0	never_IT	0	0	0	0	0	1	0	0	
	Braolecap	braolecap	angiosperm	Brassica oleraceae capitata	Angiosperms	Brassicales	Brassicaceae	Eudicots non NFN	10.1038/ncomms4930	0	0	never_IT	0	0	0	0	0	0	1	0	
	Brarap	brarap	angiosperm	Brassica rapa FPsc	Angiosperms	Brassicales	Brassicaceae	Eudicots non NFN	10.1038/s41438-018-0071-9	0	0	never_IT	0	0	0	0	0	0	1	0	1,3
	Cajcaj	cajcaj	angiosperm	Cajanus cajan	Angiosperms	Fabales	Fabaceae	NFN	10.1038/nbt.2022.	1	1	IT	0	0	0	0	0	1	0	0	
	Camsin	camsin	angiosperm	Camellia sinensis	Angiosperms	Ericales	Theaceae	Eudicots_non_NFN	[illegible]	1	0	never_IT	0	0	0	0	0	1	0	0	
	Cansat	cansat	angiosperm	Cannabis sativa	Angiosperms	Rosales	Cannabaceae	NFN	0	1	0	no_IT	0	0	0	0	0	1	0	0	
	Capann	capann	angiosperm	Capsicum annuum Zunla-1	Angiosperms	Solanales	Solanaceae	Eudicots non NFN	10.1073/pnas.1400975111	1	0	never_IT	0	0	0	0	0	1	0	0	GCF_000710875.1
	Capgra	capgra	angiosperm	Capsella grandiflora	Angiosperms	Brassicales	Brassicaceae	Eudicots non NFN	10.1038/ng.2669	0	0	never_IT	0	0	0	0	0	0	1	0	
	Caprub	caprub	angiosperm	Capsella rubella	Angiosperms	Brassicales	Brassicaceae	Eudicots non NFN	10.1038/ng.2669	0	0	never_IT	0	0	0	0	0	0	1	0	
	Carfan	carfan	angiosperm	Carpinus fangiana	Angiosperms	Fagales	Betulaceae	NFN	0	0	0	no_IT	0	0	1	0	1	0	0	0	
	Carlit	carlit	angiosperm	Carex littledaleu	Angiosperms	Cyperales	Cyperaceae	Monocots	[illegible]	0	0	never_IT	0	0	0	0	0	0	1	0	
	Carpap	carpap	angiosperm	Carica papaya	Angiosperms	Brassicales	Caricaceae	Eudicots non NFN	10.1038/nature06856	1	0	never_IT	0	0	0	0	0	1	0	0	
	Casaus	casaus	angiosperm	Castanospermum australe	Angiosperms	Fabales	Fabaceae	NFN	10.1126/science.aat1743	1	0	no_IT	0	0	0	0	0	1	0	0	
	Casgla	casgla	angiosperm	Casuarina glauca	Angiosperms	Fagales	Casuarinaceae	NFN	10.1126/science.aat1743	1	1	IT	0	0	1	0	1	1	0	0	
	Casmol	casmol	angiosperm	Castanea mollissima	Angiosperms	Fagales	Fagaceae	NFN	unpublished	1	0	no_IT	0	0	1	0	1	1	0	0	
	Cepfol	cepfol	angiosperm	Cephalotus follicularis	Angiosperms	Oxalidales	Cephalotaceae	Eudicots non NFN	10.1038/s41559-016-0059	0	0	never_IT	0	0	0	0	0	0	1	0	
	Cercan	cercan	angiosperm	Cercis canadensis	Angiosperms	Fabales	Caesalpiniaceae	NFN	10.1126/science.aat1743	1	0	no_IT	0	0	0	0	0	1	0	0	
	Cerpur	cerpur	bryophytes	Ceratodon purpureus	Mosses	Dicranales	Dicranaceae	0	[illegible]	0	0	never_IT	0	0	0	0	0	0	1	0	
	Chabra	chabra	algae_streptophyta	Chara braunii	Charophyta	Charales	Characeae	0	10.1016/j.cell.2018.06.033	0	0	never_IT	0	0	0	0	0	0	1	0	
	Chafas	chafas	angiosperm	Chamaecrista fasciculata	Angiosperms	Fabales	Caesalpiniaceae	NFN	10.1126/science.aat1743	1	1	no_IT	0	0	0	0	0	1	0	0	
	Chequi	chequi	angiosperm	Chenopodium quinoa	Angiosperms	Caryophyllales	Chenopodiaceae	Eudicots non NFN	10.1038/nature21370	0	0	never_IT	0	0	0	0	0	0	1	0	
	Chlatm	chlatm	algae_streptophyta	Chlorokybus atmophyticus	Charophyta	Chlorokybales	Chlorokybaceae	0	10.1038/s41477-019-0560-3	0	0	never_IT	0	0	0	0	0	0	1	0	
	Chlrei	chlrei	chlorophyta	Chlamydomonas reinhardtii	Chlorophyta	Chlamydomonales	Chlamydomonaceae	0	10.1126/science.1143609	0	0	never_IT	0	0	0	0	0	0	1	0	
	Chlvar	chlvar	chlorophyta	Chlorella variabilis	Chlorophyta	Chlorellales	Chlorellaceae	0	10.1105/tpc.110.076406	0	0	never_IT	0	0	0	0	0	0	1	0	
	Chocri	chocri	red_algae	Chondrus crispus	Red algae	Gigartinales	Gigartinaceae	0	10.1073/pnas.1221259110	0	0	never_IT	0	0	0	0	0	0	1	0	
	Chrzof	chrzof	chlorophyta	Chromochloris zofingiensis	Chlorophyta	Sphaeropleales	Chromochloridaceae	0	10.1073/pnas.1619928114	0	0	never_IT	0	0	0	0	0	0	1	0	
	Cicari	cicari	angiosperm	Cicer arietinum ICC4958	Angiosperms	Fabales	Fabaceae	NFN	10.1038/srep12806	1	1	IT	0	0	0	0	0	1	0	0	
	Citcle	citcle	angiosperm	Citrus clementina	Angiosperms	Sapindales	Rutaceae	Eudicots non NFN	10.1038/nbt.2906	1	0	never_IT	0	0	0	0	0	1	0	0	
	Citlan	citlan	angiosperm	Citrullus lanatus ssp vulgaris 97103	Angiosperms	Cucurbitales	Cucurbitaceae	NFN	10.1038/ng.2470	1	0	no_IT	0	0	0	0	0	1	0	0	
	Citsin	citsin	angiosperm	Citrus sinensis	Angiosperms	Sapindales	Rutaceae	Eudicots non NFN	10.1038/nbt.2906	1	0	never_IT	0	0	0	0	0	1	0	0	
	Cucargarg	cucargarg	angiosperm	[illegible]	Angiosperms	Cucurbitales	Cucurbitaceae	NFN	0	1	0	no_IT	0	0	0	0	0	1	0	0	

Cucmax	cucmax	angiosperm	Cucurbita maxima	Angiosperms	Cucurbitales	Cucurbitaceae	NFN	10.1016/j.molp.2017.09.003	1	0	no_IT	0	0	0	0	0	1	0	0	
Cucmel	cucmel	angiosperm	Cucumis melo	Angiosperms	Cucurbitales	Cucurbitaceae	NFN	10.1073/pnas.1205415109	1	0	no_IT	0	0	0	0	0	1	0	0	
Cucmos	cucmos	angiosperm	Cucurbita moschata	Angiosperms	Cucurbitales	Cucurbitaceae	NFN	10.1016/j.molp.2017.09.004	1	0	no_IT	0	0	0	0	0	1	0	0	
Cucpep	cucpep	angiosperm	Cucurbita pepo	Angiosperms	Cucurbitales	Cucurbitaceae	NFN	10.1111/pbi.12860	1	0	no_IT	0	0	0	0	0	1	0	0	
Cucsat	cucsat	angiosperm	Cucumis sativus PI183967	Angiosperms	Cucurbitales	Cucurbitaceae	NFN	10.1038/ng.2801	1	0	no_IT	0	0	0	0	0	1	0	0	
Cuscam	cuscam	angiosperm	Cuscuta campestris	Angiosperms	Solanales	Convolvulaceae	Eudicots non NFN	NCBI	0	0	never_IT	0	0	0	0	0	0	1	0	
Cyapar	cyapar	glaucophyta	Cyanophora paradoxa	Glaucophyceae	Glaucocystales	Glaucocystaceae	0	10.1126/science.1213561	0	0	never_IT	0	0	0	0	0	0	1	0	
Cycmic	cycmic	gymnosperms	Cycas micholitzii	Gymnosperms	Cycadales	Cycadaceae	0	[illegible]	1	0	never_IT	0	0	0	?	0	1	0	0	
Datglo	datglo	angiosperm	Datisca glomerata	Angiosperms	Cucurbitales	Dasticiaceae	NFN	10.1126/science.aat1743	1	1	no_IT	0	0	0	0	0	1	0	0	
Daucar	daucar	angiosperm	Daucus carota	Angiosperms	Apiales	Apiaceae	Eudicots non NFN	10.1038/ng.3565	1	0	never_IT	0	0	0	0	0	1	0	0	
Dencat	dencat	angiosperm	Dendrobium catenatum	Angiosperms	Asparagales	Orchidaceae	Monocots	10.1038/nature23897	0	0	never_IT	1	0	0	0	0	1	0	0	
Diacar	diacar	angiosperm	Dianthus caryophyllus	Angiosperms	Caryophyllales	Caryophyllaceae	Eudicots non NFN	10.1093/dnares/dst053	0	0	never_IT	0	0	0	0	0	0	1	0	
Distri	distri	angiosperm	Discaria trinervis	Angiosperms	Rosales	Rhamnaceae	NFN	10.1126/science.aat1743	1	1	no_IT	0	0	0	0	0	1	0	0	
Drydru	drydru	angiosperm	Dryas drummondii	Angiosperms	Rosales	Rosaceae	NFN	10.1126/science.aat1743	1	1	no_IT	0	0	0	0	0	1	0	0	
Dryoct	dryoct	angiosperm	Dryas octopetala	Angiosperms	Rosales	Rosaceae	NFN	10.1126/science.aat1743	0	0	no_IT	0	0	1	0	1	0	0	0	
Dunsal	dunsal	chlorophyta	Duneliella salina	Chlorophyta	Chlamydomonales	Dunaliellaceae	0	10.1128/genomeA.01105-17	0	0	never_IT	0	0	0	0	0	0	1	0	
Eurfer	eurfer	angiosperm	Euryale ferox	Angiosperms	Nymphaeales	Nymphaeaceae	0	Unpublished - NCBI	0	0	never_IT	0	0	0	0	0	0	1	0	
Eutsal	eutsal	angiosperm	Eutrema salsugineum	Angiosperms	Brassicales	Brassicaceae	Eudicots non NFN	10.3389/fpls.2013.00046	0	0	never_IT	0	0	0	0	0	0	1	0	
Fagsyl	fagsyl	angiosperm	Fagus sylvatica	Angiosperms	Fagales	Fagaceae	NFN	Unpublished	1	0	no_IT	0	0	1	0	1	1	0	0	
Faialb	faialb	angiosperm	Faidherbia albida	Angiosperms	Fabales	Fabaceae	NFN	10.1093/gigascience/giy152	1	1	IT	0	0	0	0	0	1	0	0	
Ficere	ficere	angiosperm	Ficus erecta	Angiosperms	Rosales	Moraceae	NFN	0	1	0	no_IT	0	0	0	0	0	1	0	0	
Fraana	fraana	angiosperm	Fragaria x ananassa	Angiosperms	Rosales	Rosaceae	NFN	Unpublished- https://www.rosaceae.org/organism/24345	1	0	no_IT	0	0	0	0	0	1	0	0	
Fraexc	fraexc	angiosperm	Fraxinus excelsior	Angiosperms	Lamiales	Oleaceae	Eudicots non NFN	10.1038/nature20786	1	0	never_IT	0	0	0	0	0	1	0	0	
Fraiin	fraiin	angiosperm	Fragaria iinumae	Angiosperms	Rosales	Rosaceae	NFN	0	1	0	no_IT	0	0	0	0	0	1	0	0	
Fraves	fraves	angiosperm	Fragaria vesca	Angiosperms	Rosales	Rosaceae	NFN	10.1093/gigascience/gix124	1	0	no_IT	0	0	0	0	0	1	0	0	
Galsul	galsul	red_algae	Galdieria sulphuraria	Red algae	Cyanidiales	Galdieriaceae	0	10.1126/science.1231707	0	0	never_IT	0	0	0	0	0	0	1	0	
Ginbil	ginbil	gymnosperms	Ginkgo biloba	Gymnosperms	Ginkgoales	Ginkgoaceae	0	10.5524/100613	1	0	never_IT	0	0	0	0	0	1	0	0	
Glymax	glymax	angiosperm	Glycine max	Angiosperms	Fabales	Fabaceae	NFN	10.1038/nature08670	1	1	IT	0	0	0	0	0	1	0	0	
Glysoj	glysoj	angiosperm	Glycine soja	Angiosperms	Fabales	Fabaceae	NFN	0	1	1	IT	0	0	0	0	0	1	0	0	
Gnemon	gnemon	gymnosperms	Gnetum montanum	Gymnosperms	Gnetales	Gnetaceae	0	1KP / 10.5061/dryad.0vm37	0	0	never_IT	0	0	1	0	1	0	0	0	
Gosrai	gosrai	angiosperm	Gossypium raimondii	Angiosperms	Malvales	Malvaceae	Eudicots non NFN	10.1038/nature11798	1	0	never_IT	0	0	0	0	0	1	0	0	
Helann	helann	angiosperm	Helianthus annuus	Angiosperms	Asterales	Asteraceae	Eudicots non NFN	10.1038/nature22380	1	0	never_IT	0	0	0	0	0	1	0	0	
Hevbra	hevbra	angiosperm	Hevea brasiliensis	Angiosperms	Malpighiales	Euphorbiaceae	Eudicots non NFN	0	1	0	never_IT	0	0	0	0	0	1	0	0	
Horvul	horvul	angiosperm	Hordeum vulgare	Angiosperms	Poales	Poaceae	Monocots	10.1038/nature22043/10.1038/sdata.2017.44	1	0	never_IT	0	0	0	0	0	1	0	0	
HorvulGPv1r1	horvulGPv1r1	angiosperm	Hordeum vulgare	Angiosperms	Poales	Poaceae	Monocots	Unpublished	1	0	never_IT	0	0	0	0	0	1	0	0	
Humlup	humlup	angiosperm	Humulus lupulus	Angiosperms	Rosales	Cannabaceae	NFN	10.1093/pcp/pcu169	1	0	no_IT	0	0	0	0	0	1	0	0	
Jatcur	jatcur	angiosperm	Jatropha curcas	Angiosperms	Malpighiales	Euphorbiaceae	Eudicots non NFN	0	1	0	never_IT	0	0	0	0	0	1	0	0	
Jugreg	jugreg	angiosperm	Juglans regia	Angiosperms	Fagales	Juglandaceae	NFN	10.1111/tpj.13207	1	0	no_IT	0	0	1	0	1	1	0	0	
Klenit	klenit	algae_streptophyta	Klebsormidium nitens	Charophyta	Klebsormidiales	Klebsormidiaceae	0	10.1038/ncomms4978	0	0	never_IT	0	0	0	0	0	0	1	0	
Labpur	labpur	angiosperm	Lablab purpureus	Angiosperms	Fabales	Fabaceae	NFN	10.1093/gigascience/giy152	1	1	IT	0	0	0	0	0	1	0	0	
Lagsic	lagsic	angiosperm	Lagenaria siceraria	Angiosperms	Cucurbitales	Cucurbitaceae	NFN	10.1111/tpj.13722	1	0	no_IT	0	0	0	0	0	1	0	0	
Linusi	linusi	angiosperm	Linum usitatissimum	Angiosperms	Malpighiales	Linaceae	Eudicots non NFN	0	1	0	never_IT	0	0	0	0	0	1	0	0	
LotjapGifu	lotjapGifu	angiosperm	Lotus japonicus	Angiosperms	Fabales	Fabaceae	NFN	10.1101/2020.05.29.124313	1	1	IT	0	0	0	0	0	1	0	0	1,2
Lupalb	lupalb	angiosperm	Lupinus albus	Angiosperms	Fabales	Fabaceae	NFN	10.1038/s41467-019-14197-9	0	1	no_IT	0	0	0	0	0	1	0	0	
Lupang	lupang	angiosperm	Lupinus angustifolius	Angiosperms	Fabales	Fabaceae	NFN	10.1111/pbi.12615	0	1	no_IT	0	0	0	0	0	1	0	0	
Malbac	malbac	angiosperm	Malus baccata	Angiosperms	Rosales	Rosaceae	NFN	0	1	0	no_IT	0	0	0	0	0	1	0	0	
Maldom	maldom	angiosperm	Malus domestica	Angiosperms	Rosales	Rosaceae	NFN	Unpublished - rosaceae.org	1	0	no_IT	0	0	0	0	0	1	0	0	1,1
Manesc	manesc	angiosperm	Manihot esculenta	Angiosperms	Malpighiales	Euphorbiaceae	Eudicots non NFN	10.1038/nbt.3535	1	0	never_IT	0	0	0	0	0	1	0	0	
Marinf	marinf	bryophytes	Marchantia inflexa	Liverworts	Marchantiales	Marchantiaceae	0	10.1038/s41598-019-45039-9	1	0	never_IT	0	0	0	0	0	1	0	0	
Marpal	marpal	bryophytes	Marchantia paleacea	Liverworts	Marchantiales	Marchantiaceae	0	Unpublished	1	0	never_IT	0	0	0	0	0	1	0	0	
Marpalillu	marpalillu	bryophytes	Marchantia paleacea	Liverworts	Marchantiales	Marchantiaceae	0	10.1038/s41477-020-0613-7	1	0	never_IT	0	0	0	0	0	1	0	0	
Medtru	medtru	angiosperm	Medicago truncatula	Angiosperms	Fabales	Fabaceae	NFN	10.1038/s41477-018-0286-7	1	1	IT	0	0	0	0	0	1	0	0	
Mesend	mesend	algae_streptophyta	Mesotaenium endlicherianum	Charophyta	Zygnemales	Zygnematophyceae	0	10.1016/j.cell.2019.10.019	0	0	never_IT	0	0	0	0	0	0	1	0	
Mesvir	mesvir	algae_streptophyta	Mesostigma viride	Charophyta	Mesostigmatales	Mesostigmataceae	0	10.1038/s41477-019-0560-3	0	0	never_IT	0	0	0	0	0	0	1	0	
Micpus1545	micpus1545	chlorophyta	Micromonas pusillaCCMP1545	Chlorophyta	Mamiellales	Mamiellaceae	0	10.1126/science.1167222	0	0	never_IT	0	0	0	0	0	0	1	0	
MicpusNOUM17	micpusNOUM17	chlorophyta	Micromonas pusillaCCMP1546	Chlorophyta	Mamiellales	Mamiellaceae	0	#N/A	0	0	0	0	0	0	0	0	0	1	0	0
Mimpud	mimpud	angiosperm	Mimosa pudica	Angiosperms	Fabales	Fabaceae	NFN	Unpublished	1	1	IT	0	0	0	0	0	1	0	0	
Momcha	momcha	angiosperm	Momordica charantia	Angiosperms	Cucurbitales	Cucurbitaceae	NFN	Unpublished - NCBI	1	0	no_IT	0	0	0	0	0	1	0	0	
Monneg	monneg	chlorophyta	Monoraphidium neglectum	Chlorophyta	Sphaeropleales	Selenastraceae	0	10.1186/1471-2164-14-926	0	0	never_IT	0	0	0	0	0	0	1	0	
Mornot	mornot	angiosperm	Morus notabilis	Angiosperms	Rosales	Moraceae	NFN	10.1038/ncomms3445	1	0	no_IT	0	0	0	0	0	1	0	0	

Mucpru	mucpru	angiosperm	Mucuna pruriens	Angiosperms	Fabales	Fabaceae	NFN	0	1	1	IT	0	0	0	0	0	1	0	0	
Musacu	musacu	angiosperm	Musa acuminata	Angiosperms	Zingiberales	Zingiberaceae	Monocots	10.1093/database/bat035	1	0	never_IT	0	0	0	0	0	1	0	0	
Nelnuc	nelnuc	angiosperm	Nelumbo nucifera	Angiosperms	Proteales	Nelumbonaceae	BasalEudicots	10.1186/gb-2013-14-5-r41	0	0	never_IT	0	0	0	0	0	0	1	0	
Nicben	nicben	angiosperm	Nicotiana benthamiana	Angiosperms	Solanales	Solanaceae	Eudicots non NFN	10.1094/MPMI-06-12-0148-TA	1	0	never_IT	0	0	0	0	0	1	0	0	
Nissch	nissch	angiosperm	Nissolia schottii	Angiosperms	Fabales	Fabaceae	NFN	10.1126/science.aat1743	1	0	no_IT	0	0	0	0	0	1	0	0	
Nymcol	nymcol	angiosperm	Nymphea colorata	Angiosperms	Nymphaeales	Nymphaeaceae	0	[illegible]	0	0	never_IT	0	0	0	0	0	0	1	0	
Orysat	orysat	angiosperm	Oryza sativa	Angiosperms	Poales	Poaceae	Monocots	10.1093/nar/gkl976	1	0	never_IT	0	0	0	0	0	1	0	0	
Ostluc	ostluc	chlorophyta	Ostreococcus lucimarinus	Chlorophyta	Mamiellales	Mamiellaceae	0	10.1073/pnas.0611046104	0	0	never_IT	0	0	0	0	0	0	1	0	
Osttau	osttau	chlorophyta	Ostreococcus tauri	Chlorophyta	Mamiellales	Mamiellaceae	0	10. 1186/1471- 2164- 15- 1103/10. 1073/pnas. 0611046104	0	0	never_IT	0	0	0	0	0	0	1	0	
Parand	parand	angiosperm	Parasponia andersonii	Angiosperms	Rosales	Cannabaceae	NFN	10.1073/pnas.1721395115	1	1	no_IT	0	0	0	0	0	1	0	0	
Penmar	penmar	algae_streptophyta	Penium margaritaceum	Charophyta	Desmidiales	Peniaceae	0	10.1016/j.cell.2020.04.019	0	0	never_IT	0	0	0	0	0	0	1	0	
Petaxi	petaxi	angiosperm	Petunia axillaris	Angiosperms	Solanales	Solanaceae	Eudicots non NFN	10.1038/nplants.2016.74	1	0	never_IT	0	0	0	0	0	1	0	0	
Phaequ	phaequ	angiosperm	Phalaenopsis equestris	Angiosperms	Asparagales	Orchidaceae	Monocots	10.1038/nature23897	0	0	never_IT	1	0	0	0	0	1	0	0	
Phavul	phavul	angiosperm	Phaseolus vulgaris	Angiosperms	Fabales	Fabaceae	NFN	10.1038/ng.3008	1	1	IT	0	0	0	0	0	1	0	0	
Phypat	phypat	bryophytes	Physcomitrella patens	Mosses	Funariales	Funariaceae	0	10.1111/tpj.13801	0	0	never_IT	0	0	0	0	0	0	1	0	
Picabi	picabi	gymnosperms	Picea abies	Gymnosperms	Pinales	Pinaceae	0	10.1038/nature12211	0	0	never_IT	0	0	1	0	1	0	0	0	
Picgla	picgla	gymnosperms	Picea glauca	Gymnosperms	Pinales	Pinaceae	0	10.1093/bioinformatics/btt178	0	0	never_IT	0	0	1	0	1	0	0	0	
Picsit	picsit	gymnosperms	Picea sitchensis	Gymnosperms	Pinales	Pinaceae	0	Unpublished - gymno-plaza	0	0	never_IT	0	0	1	0	1	0	0	0	
Pinpin	pinpin	gymnosperms	Pinus pinaster	Gymnosperms	Pinales	Pinaceae	0	Unpublished - gymno-plaza	0	0	never_IT	0	0	1	0	1	0	0	0	
Pinsyl	pinsyl	gymnosperms	Pinus sylvestris	Gymnosperms	Pinales	Pinaceae	0	Unpublished - gymno-plaza	0	0	never_IT	0	0	1	0	1	0	0	0	
Pintae	pintae	gymnosperms	Pinus taeda	Gymnosperms	Pinales	Pinaceae	0	10.1534/genetics.113.159715	0	0	never_IT	0	0	1	0	1	0	0	0	
Pissat	pissat	angiosperm	Pissum sativum	Angiosperms	Fabales	Fabaceae	NFN	10.1038/s41588-019-0480-1	1	1	IT	0	0	0	0	0	1	0	0	
Popalb	popalb	angiosperm	Populus alba	Angiosperms	Malpighiales	Salicaceae	Eudicots non NFN	0	1	0	never_IT	0	0	1	0	1	1	0	0	
Popeup	popeup	angiosperm	Populus euphratica	Angiosperms	Malpighiales	Salicaceae	Eudicots non NFN	0	1	0	never_IT	0	0	1	0	1	1	0	0	
Poptri	poptri	angiosperm	Populus trichocarpa	Angiosperms	Malpighiales	Salicaceae	Eudicots non NFN	10.1126/science.1128691	1	0	never_IT	0	0	1	0	1	1	0	0	
Porpur	porpur	red_algae	Porphyridium purpureum	Red algae	Porphyridiales	Porphyridiaceae	0	10.1038/ncomms2931	0	0	never_IT	0	0	0	0	0	0	1	0	
Porumb	porumb	red_algae	Porphyra umbilicalis	Red algae	Bangiales	Bangiaceae	0	10.1073/pnas.1703088114	0	0	never_IT	0	0	0	0	0	0	1	0	
Potmic	potmic	angiosperm	Potentilla micrantha	Angiosperms	Rosales	Rosaceae	NFN	10.1093/gigascience/giy010	1	0	no_IT	0	0	0	0	0	1	0	0	
Proalb	proalb	angiosperm	Prosopis alba	Angiosperms	Fabales	Fabaceae	NFN	0	1	1	IT	0	0	0	0	0	1	0	0	
Pruavi	pruavi	angiosperm	Prunus avium	Angiosperms	Rosales	Rosaceae	NFN	10.1093/dnares/dsx020	1	0	no_IT	0	0	0	0	0	1	0	0	
Prudul	prudul	angiosperm	Prunus dulcis	Angiosperms	Rosales	Rosaceae	NFN	10.1111/tpj.14538	1	0	no_IT	0	0	0	0	0	1	0	0	2.0
Prumum	prumum	angiosperm	Prunus mume	Angiosperms	Rosales	Rosaceae	NFN	Unpublished - NCBI	1	0	no_IT	0	0	0	0	0	1	0	0	
Pruper	pruper	angiosperm	Prunus persica	Angiosperms	Rosales	Rosaceae	NFN	10.1038/ng.2586	1	0	no_IT	0	0	0	0	0	1	0	0	
Pruyed	pruyed	angiosperm	Prunus yeodensis	Angiosperms	Rosales	Rosaceae	NFN	0	1	0	no_IT	0	0	0	0	0	1	0	0	
Psemen	psemen	gymnosperms	Pseudotsuga menziesii	Gymnosperms	Pinales	Pinaceae	0	10.1534/g3.117.300078	0	0	never_IT	0	0	1	0	1	0	0	0	
Purtri	purtri	angiosperm	Purshia tridentata	Angiosperms	Rosales	Rosaceae	NFN	Unpublished	1	1	IT	0	0	1	0	1	1	0	0	
Pyrbet	pyrbet	angiosperm	Pyrus betulifolia	Angiosperms	Rosales	Rosaceae	NFN	0	1	0	no_IT	0	0	0	0	0	1	0	0	
Pyrbre	pyrbre	angiosperm	Pyrus x bretschneideri	Angiosperms	Rosales	Rosaceae	NFN	Unpublished - NCBI	1	0	no_IT	0	0	0	0	0	1	0	0	
Pyrcom	pyrcom	angiosperm	Pyrus communis	Angiosperms	Rosales	Rosaceae	NFN	10.1371/journal.pone.0092644	1	0	no_IT	0	0	0	0	0	1	0	0	
Pyryez	pyryez	red_algae	Pyropia yezoensis	Red algae	Bangiales	Bangiaceae	0	10.1371/journal.pone.0057122	0	0	never_IT	0	0	0	0	0	0	1	0	
Quelob	quelob	angiosperm	Quercus lobata	Angiosperms	Fagales	Fagaceae	NFN	0	1	0	no_IT	0	0	1	0	1	1	0	0	
Querob	querob	angiosperm	Quercus robur	Angiosperms	Fagales	Fagaceae	NFN	10.1111/1755-0998.12425	1	0	no_IT	0	0	1	0	1	1	0	0	
Rharub	rharub	angiosperm	Rhamnella rubrinervis	Angiosperms	Rosales	Rhamnaceae	NFN	0	1	0	no_IT	0	0	0	0	0	1	0	0	
Rhowil	rhowil	angiosperm	Rhododendron williamsianum	Angiosperms	Ericales	Ericaceae	Eudicots non NFN	10.1093/gbe/evz245	0	0	never_IT	0	1	0	0	0	1	0	0	
Riccom	riccom	angiosperm	Ricinus communis	Angiosperms	Malpighiales	Euphorbiaceae	Eudicots non NFN	10.1038/nbt.1674	1	0	never_IT	0	0	0	0	0	1	0	0	GCF_000151685. 1
Roschi	roschi	angiosperm	Rosa chinensis	Angiosperms	Rosales	Rosaceae	NFN	10.1038/s41588-018-0110-3	1	0	no_IT	0	0	0	0	0	1	0	0	
Rubocc	rubocc	angiosperm	Rubus occidentalis	Angiosperms	Rosales	Rosaceae	NFN	10.1111/tpj.13215	1	0	no_IT	0	0	0	0	0	1	0	0	
Salcuc	salcuc	ferns	Salvinia cucullata	Leptosporangiate	Salviniales	Salviniaceae	0	10.1038/s41477-018-0188-9	0	0	never_IT	0	0	0	0	0	0	1	0	
Sclbir	sclbir	angiosperm	Sclerocarya birrea	Angiosperms	Sapindales	Anacardiaceae	Eudicots non NFN	10.1093/gigascience/giy152	1	0	never_IT	0	0	0	0	0	1	0	0	
Sellep	sellep	lycophytes	Selaginella lepidophylla	Lycophytes	Selaginellales	Selaginellaceae	0	10.1038/s41467-017-02546-5	1	0	never_IT	0	0	0	0	0	1	0	0	
Selmoe	selmoe	lycophytes	Selaginella moellendorffii	Lycophytes	Selaginellales	Selaginellaceae	0	10.1126/science.1203810	1	0	never_IT	0	0	0	0	0	1	0	0	
Setita	setita	angiosperm	Setaria italica	Angiosperms	Poales	Poaceae	Monocots	10.1038/nbt.2196	1	0	never_IT	0	0	0	0	0	1	0	0	
Sollyc	sollyc	angiosperm	Solanum lycopersicum	Angiosperms	Solanales	Solanaceae	Eudicots non NFN	10.1038/nature11119	1	0	never_IT	0	0	0	0	0	1	0	0	
Solpen	solpen	angiosperm	Solanum pennellii	Angiosperms	Solanales	Solanaceae	Eudicots non NFN	10.1038/ng.3046	1	0	never_IT	0	0	0	0	0	1	0	0	
Sorbic	sorbic	angiosperm	Sorghum bicolor	Angiosperms	Poales	Poaceae	Monocots	10.1111/tpj.13781	1	0	never_IT	0	0	0	0	0	1	0	0	
Spasub	spasub	angiosperm	Spatholobus suberectus	Angiosperms	Fabales	Fabaceae	NFN	0	1	1	IT	0	0	0	0	0	1	0	0	
Sphfal	sphfal	bryophytes	Sphagnum fallax	Mosses	Sphagnales	Sphagnaceae	0	Unpublished - Phytozome	0	0	never_IT	0	0	0	0	0	0	1	1	0,5
Spimus	spimus	algae_streptophyta	Spirogloea muscicola	Charophyta	Zygnemales	Zygnematophyceae	0	10.1016/j.cell.2019.10.019	0	0	never_IT	0	0	0	0	0	0	1	0	
Spiole	spiole	angiosperm	Spinacia oleracea	Angiosperms	Caryophyllales	Amaranthaceae	Eudicots non NFN	Unpublished - bvseq.molgen.mpg.de	0	0	never_IT	0	0	0	0	0	0	1	0	1.0
Spipol	spipol	angiosperm	Spirodela polyrhiza	Angiosperms	Alismatales	Araceae	Monocots	10.1038/ncomms4311	0	0	never_IT	0	0	0	0	0	0	1	0	

	Tarhas	tarhas	angiosperm	Tarenaya hassleriana	Angiosperms	Brassicales	Cleomaceae	Eudicots non NFN	10.1105/tpc.113.113480	0	0	never_IT	0	0	0	0	0	0	1	0	
	Thecac	thecac	angiosperm	Theobroma cacao	Angiosperms	Malvales	Malvaceae	Eudicots non NFN	10.1186/gb-2013-14-6-r53	1	0	never_IT	0	0	0	0	0	1	0	0	
	Treori	treori	angiosperm	Trema orientalis	Angiosperms	Rosales	Cannabaceae	NFN	10.1073/pnas.1721395115	1	0	no_IT	0	0	0	0	0	1	0	0	
	Tripra	tripra	angiosperm	Trifolium pratense	Angiosperms	Fabales	Fabaceae	NFN	10.1038/srep17394	1	1	IT	0	0	0	0	0	1	0	0	
	Trisub	trisub	angiosperm	Trifolium subterraneum	Angiosperms	Fabales	Fabaceae	NFN	Unpublished - NCBI	1	1	IT	0	0	0	0	0	1	0	0	
	Utrgib	utrgib	angiosperm	Utricularia gibba	Angiosperms	Lamiales	Lentibulariaceae	0	10.1073/pnas.1702072114	0	0	never_IT	0	0	0	0	0	0	1	0	
	Utrren	utrren	angiosperm	Utricularia reniformis	Angiosperms	Lamiales	Lentibulariaceae	0	10.3390/ijms21010003	0	0	never_IT	0	0	0	0	0	0	1	0	
	Vigang	vigang	angiosperm	Vigna angularis	Angiosperms	Fabales	Fabaceae	NFN	10.1038/srep080669	1	1	IT	0	0	0	0	0	1	0	0	
	Vigrad	vigrad	angiosperm	Vigna radiata	Angiosperms	Fabales	Fabaceae	NFN	10.1038/ncomms6443	1	1	IT	0	0	0	0	0	1	0	0	
	Vigsub	vigsub	angiosperm	Vigna subterranea	Angiosperms	Fabales	Fabaceae	NFN	10.1093/gigascience/giy152	1	1	IT	0	0	0	0	0	1	0	0	
	Vigung	vigung	angiosperm	Vigna unguiculata	Angiosperms	Fabales	Fabaceae	NFN	10.1111/tpj.14349	1	1	IT	0	0	0	0	0	1	0	0	
	Volcar	volcar	chlorophyta	Volvox carteri	Chlorophyta	Chlamydomonales	Volvocaceae	0	10.1126/science.1188800	0	0	never_IT	0	0	0	0	0	0	1	0	
	Zeamay	zeamay	angiosperm	Zea mays PH207	Angiosperms	Poales	Poaceae	Monocots	10.1105/tpc.16.00353	1	0	never_IT	0	0	0	0	0	1	0	0	
	Zizjuj	zizjuj	angiosperm	Ziziphus jujuba cv. Dongzao	Angiosperms	Rosales	Rhamnaceae	NFN	10.1038/ncomms6315	1	0	no_IT	0	0	0	0	0	1	0	0	
	Zosmar	zosmar	angiosperm	Zostera marina	Angiosperms	Alismatales	Zosteraceae	Monocots	10.1038/nature16548	0	0	never_IT	0	0	0	0	0	0	1	0	
	Zygcir	zygcir	algae_streptophyta	Zygnema circumcarinatum SAG698	Charophyta	Zygnematales	Zygnemataceae	0	Unpublished	0	0	0	0	0	0	0	0	0	1		
in_db	Rhosim	rhosim	angiosperm	Rhododendron simsii	Angiosperms	Ericales	Ericaceae	Eudicots non NFN	10.1038/s41467-020-18771-4	0	0	never_IT	0	1?	0	0	0	1	0	0	
in_db	Triaes	triaes	angiosperm	Triticum aestivum IWGSC_v1.1_HC_20170706	Angiosperms	Poales	Poaceae	Monocots	10.1093/gigascience/gix097	1	0	never_IT	0	0	0	0	0	1	0	0	
in_db	Aeseve	aeseve	angiosperm	Aeschynomene evenia	Angiosperms	Fabales	Fabaceae	NFN	10.1038/s41467-021-21094-7	1	1	no_IT	0	0	0	0	0	1	0	0	v1

Species_abbreviation	Species_name	Group
Abrpre	*Abrus precatorius*	Angiosperms
Aeseve	*Aeschynomene evenia*	Angiosperms
Alnglu	*Alnus glutinosa*	Angiosperms
Amahyp	*Amaranthus hypochondriacus*	Angiosperms
Ambtri	*Amborella trichopoda*	Angiosperms
Anacom	*Ananas comosus*	Angiosperms
AntagrBONN	*Anthoceros agrestis*	Hornworts
AntagrOXF	*Anthoceros agrestis*	Hornworts
Antpun	*Anthoceros punctatus*	Hornworts
Aposhe	*Apostasia shenzhenica*	Angiosperms
Aqucoe	*Aquilegia coerulea*	Angiosperms
Aradur	*Arachis duranensis*	Angiosperms
Arahal	*Arabidopsis halleri*	Angiosperms
Arahyp	*Arachis hypogaea*	Angiosperms
Araipa	*Arachis ipaensis*	Angiosperms
Aralyr	*Arabidopsis lyrata*	Angiosperms
Aratha	*Arabidopsis thaliana*	Angiosperms
Auxpro	*Auxenochlorella protothecoides*	Chlorophyta
Azofil	*Azolla filiculoides*	Leptosporangiate
Batpra	*Bathycoccus prasinos RCC1105*	Chlorophyta
Begfuc	*Begonia fuchsioides*	Angiosperms
Benhis	*Benincasa hispida*	Angiosperms
Betpat	*Beta patula*	Angiosperms
Betpen	*Betula pendula*	Angiosperms
Betvul	*Beta vulgaris ssp vulgaris KWS2320*	Angiosperms
Boestr	*Boechera stricta*	Angiosperms
Botbra	*Botryococcus braunii*	Chlorophyta
Bradis	*Brachypodium distachyon*	Angiosperms
Braolecap	*Brassica oleraceae capitata*	Angiosperms
Brarap	*Brassica rapa FPsc*	Angiosperms
Cajcaj	*Cajanus cajan*	Angiosperms
Camsin	*Camellia sinensis*	Angiosperms
Cansat	*Cannabis sativa*	Angiosperms
Capann	*Capsicum annuum Zunla-1*	Angiosperms
Capgra	*Capsella grandiflora*	Angiosperms
Caprub	*Capsella rubella*	Angiosperms
Carfan	*Carpinus fangiana*	Angiosperms
Carlit	*Carex littledaleu*	Angiosperms
Carpap	*Carica papaya*	Angiosperms
Casaus	*Castanospermum australe*	Angiosperms
Casgla	*Casuarina glauca*	Angiosperms
Casmol	*Castanea mollissima*	Angiosperms
Cepfol	*Cephalotus follicularis*	Angiosperms
Cercan	*Cercis canadensis*	Angiosperms
Cerpur	*Ceratodon purpureus*	Mosses
Chabra	*Chara braunii*	Charophyta
Chafas	*Chamaecrista fasciculata*	Angiosperms
Chequi	*Chenopodium quinoa*	Angiosperms
Chlatm	*Chlorokybus atmophyticus*	Charophyta
Chlrei	*Chlamydomonas reinhardtii*	Chlorophyta
Chlvar	*Chlorella variabilis*	Chlorophyta

Chocri	*Chondrus crispus*	Red algae
Chrzof	*Chromochloris zofingiensis*	Chlorophyta
Cicari	*Cicer arietinum ICC4958*	Angiosperms
Citcle	*Citrus clementina*	Angiosperms
Citlan	*Citrullus lanatus ssp vulgaris 97103*	Angiosperms
Citsin	*Citrus sinensis*	Angiosperms
Cucargarg	*Cucurbita argyrosperma subsp. agyrosperma*	Angiosperms
Cucmax	*Cucurbita maxima*	Angiosperms
Cucmel	*Cucumis melo*	Angiosperms
Cucmos	*Cucurbita moschata*	Angiosperms
Cucpep	*Cucurbita pepo*	Angiosperms
Cucsat	*Cucumis sativus PI183967*	Angiosperms
Cuscam	*Cuscuta campestris*	Angiosperms
Cyapar	*Cyanophora paradoxa*	Glaucophyceae
Cycmic	*Cycas micholitzii*	Gymnosperms
Datglo	*Datisca glomerata*	Angiosperms
Daucar	*Daucus carota*	Angiosperms
Dencat	*Dendrobium catenatum*	Angiosperms
Diacar	*Dianthus caryophyllus*	Angiosperms
Distri	*Discaria trinervis*	Angiosperms
Drydru	*Dryas drummondii*	Angiosperms
Dunsal	*Duneliella salina*	Chlorophyta
Eurfer	*Euryale ferox*	Angiosperms
Eutsal	*Eutrema salsugineum*	Angiosperms
Faialb	*Faidherbia albida*	Angiosperms
Ficere	*Ficus erecta*	Angiosperms
Fraana	*Fragaria x ananassa*	Angiosperms
Fraexc	*Fraxinus excelsior*	Angiosperms
Fraiin	*Fragaria iinumae*	Angiosperms
Fraves	*Fragaria vesca*	Angiosperms
Galsul	*Galdieria sulphuraria*	Red algae
Ginbil	*Ginkgo biloba*	Gymnosperms
Glymax	*Glycine max*	Angiosperms
Glysoj	*Glycine soja*	Angiosperms
Gnemon	*Gnetum montanum*	Gymnosperms
Gosrai	*Gossypium raimondii*	Angiosperms
Helann	*Helianthus annuus*	Angiosperms
Hevbra	*Hevea brasiliensis*	Angiosperms
Horvul	*Hordeum vulgare*	Angiosperms
Humlup	*Humulus lupulus*	Angiosperms
Jatcur	*Jatropha curcas*	Angiosperms
Jugreg	*Juglans regia*	Angiosperms
Klenit	*Klebsormidium nitens*	Charophyta
Labpur	*Lablab purpureus*	Angiosperms
Lagsic	*Lagenaria siceraria*	Angiosperms
Linusi	*Linum usitatissimum*	Angiosperms
LotjapGifu	*Lotus japonicus*	Angiosperms
Lupalb	*Lupinus albus*	Angiosperms
Lupang	*Lupinus angustifolius*	Angiosperms
Malbac	*Malus baccata*	Angiosperms
Maldom	*Malus domestica*	Angiosperms
Manesc	*Manihot esculenta*	Angiosperms

Marinf	*Marchantia inflexa*	Liverworts
Marpalillu	*Marchantia paleacea*	Liverworts
Medtru	*Medicago truncatula*	Angiosperms
Mesend	*Mesotaenium endlicherianum*	Charophyta
Mesvir	*Mesostigma viride*	Charophyta
Micpus1545	*Micromonas pusilla CCMP1545*	Chlorophyta
Mimpud	*Mimosa pudica*	Angiosperms
Momcha	*Momordica charantia*	Angiosperms
Monneg	*Monoraphidium neglectum*	Chlorophyta
Mornot	*Morus notabilis*	Angiosperms
Mucpru	*Mucuna pruriens*	Angiosperms
Musacu	*Musa acuminata*	Angiosperms
Nelnuc	*Nelumbo nucifera*	Angiosperms
Nicben	*Nicotiana benthamiana*	Angiosperms
Nissch	*Nissolia schottii*	Angiosperms
Nymcol	*Nymphea colorata*	Angiosperms
Orysat	*Oryza sativa*	Angiosperms
Ostluc	*Ostreococcus lucimarinus*	Chlorophyta
Osttau	*Ostreococcus tauri*	Chlorophyta
Parand	*Parasponia andersonii*	Angiosperms
Penmar	*Penium margaritaceum*	Charophyta
Petaxi	*Petunia axillaris*	Angiosperms
Phaequ	*Phalaenopsis equestris*	Angiosperms
Phavul	*Phaseolus vulgaris*	Angiosperms
Phypat	*Physcomitrella patens*	Mosses
Picabi	*Picea abies*	Gymnosperms
Picgla	*Picea glauca*	Gymnosperms
Picsit	*Picea sitchensis*	Gymnosperms
Pinpin	*Pinus pinaster*	Gymnosperms
Pinsyl	*Pinus sylvestris*	Gymnosperms
Pintae	*Pinus taeda*	Gymnosperms
Pissat	*Pisum sativum*	Angiosperms
Popalb	*Populus alba*	Angiosperms
Popeup	*Populus euphratica*	Angiosperms
Poptri	*Populus trichocarpa*	Angiosperms
Porpur	*Porphyridium purpureum*	Red algae
Porumb	*Porphyra umbilicalis*	Red algae
Potmic	*Potentilla micrantha*	Angiosperms
Proalb	*Prosopis alba*	Angiosperms
Pruavi	*Prunus avium*	Angiosperms
Prudul	*Prunus dulcis*	Angiosperms
Prumum	*Prunus mume*	Angiosperms
Pruper	*Prunus persica*	Angiosperms
Pruyed	*Prunus yeodensis*	Angiosperms
Psemen	*Pseudotsuga menziesii*	Gymnosperms
Pyrbet	*Pyrus betulifolia*	Angiosperms
Pyrbre	*Pyrus x bretschneideri*	Angiosperms
Pyrcom	*Pyrus communis*	Angiosperms
Pyryez	*Pyropia yezoensis*	Red algae
Quelob	*Quercus lobata*	Angiosperms
Querob	*Quercus robur*	Angiosperms
Rharub	*Rhamnella rubrinervis*	Angiosperms

Rhosim	*Rhododendron simsii*	Angiosperms
Rhowil	*Rhododendron williamsianum*	Angiosperms
Riccom	*Ricinus communis*	Angiosperms
Roschi	*Rosa chinensis*	Angiosperms
Rubocc	*Rubus occidentalis*	Angiosperms
Salcuc	*Salvinia cucullata*	Leptosporangiate
Sclbir	*Sclerocarya birrea*	Angiosperms
Sellep	*Selaginella lepidophylla*	Lycophytes
Selmoe	*Selaginella moellendorffii*	Lycophytes
Setita	*Setaria italica*	Angiosperms
Sollyc	*Solanum lycopersicum*	Angiosperms
Solpen	*Solanum pennellii*	Angiosperms
Sorbic	*Sorghum bicolor*	Angiosperms
Spasub	*Spatholobus suberectus*	Angiosperms
Sphfal	*Sphagnum fallax*	Mosses
Spimus	*Spirogloea muscicola*	Charophyta
Spiole	*Spinacia oleracea*	Angiosperms
Spipol	*Spirodela polyrhiza*	Angiosperms
Tarhas	*Tarenaya hassleriana*	Angiosperms
Thecac	*Theobroma cacao*	Angiosperms
Treori	*Trema orientalis*	Angiosperms
Triaes	*Triticum aestivum IWGSC_v1.1_HC_20170706*	Angiosperms
Tripra	*Trifolium pratense*	Angiosperms
Trisub	*Trifolium subterraneum*	Angiosperms
Utrgib	*Utricularia gibba*	Angiosperms
Utrren	*Utricularia reniformis*	Angiosperms
Vigang	*Vigna angularis*	Angiosperms
Vigrad	*Vigna radiata*	Angiosperms
Vigsub	*Vigna subterranea*	Angiosperms
Vigung	*Vigna unguiculata*	Angiosperms
Volcar	*Volvox carteri*	Chlorophyta
Zeamay	*Zea mays PH207*	Angiosperms
Zizjuj	*Ziziphus jujuba cv. Dongzao*	Angiosperms
Zosmar	*Zostera marina*	Angiosperms

Order	Family	Source	AMS	RNS	InfectionThread
Fabales	Fabaceae	NCBI	1	1	IT
Fabales	Fabaceae	10.1038/s414	1	1	no_IT
Fagales	Betulaceae	10.1126/scier	1	1	IT
Caryophyllales	Amaranthaceae	10.3835/plan	0	0	never_IT
Amborellales	Amborellaceae	10.1126/scier	1	0	never_IT
Poales	Bromeliaceae	10.1038/ng.3	1	0	never_IT
Anthocerotales	Anthocerataceae	10.1038/s414	1	0	never_IT
Anthocerotales	Anthocerataceae	10.1038/s414	1	0	never_IT
Anthocerotales	Anthocerataceae	10.1038/s414	1	0	never_IT
Asparagales	Orchidaceae	10.1038/natu	0	0	never_IT
Ranunculales	Ranunculaceae	10.7554/eLife	1	0	never_IT
Fabales	Fabaceae	10.1038/ng.3	1	1	no_IT
Brassicales	Brassicaceae	10.5061/drya	0	0	never_IT
Fabales	Fabaceae	10.25739/hb!	1	1	no_IT
Fabales	Fabaceae	10.1038/ng.3	1	1	no_IT
Brassicales	Brassicaceae	10.1038/ng.8	0	0	never_IT
Brassicales	Brassicaceae	10.1093/nar/	0	0	never_IT
Chlorellales	Chlorellaceae	10.1186/147	0	0	never_IT
Salviniales	Salviniaceae	10.1038/s414	0	0	never_IT
Mamiellales	Bathycoccaceae	10.1186/gb-2	0	0	never_IT
Cucurbitales	Begoniaceae	10.1126/scier	1	0	no_IT
Cucurbitales	Cucurbitaceae	10.1038/s414	1	0	no_IT
Caryophyllales	Amaranthaceae	10.1111/tpj.1	0	0	never_IT
Fagales	Fagaceae	NCBI	0	0	no_IT
Caryophyllales	Amaranthaceae	10.1101/202	0	0	never_IT
Brassicales	Brassicaceae	Phytozome	0	0	never_IT
Trebouxiales	Botryococcaceae	Phytozome	0	0	never_IT
Poales	Poaceae	10.1038/natu	1	0	never_IT
Brassicales	Brassicaceae	10.1038/ncor	0	0	never_IT
Brassicales	Brassicaceae	10.1038/s414	0	0	never_IT
Fabales	Fabaceae	10.1038/nbt.	1	1	IT
Ericales	Theaceae	NCBI	1	0	never_IT
Rosales	Cannabaceae	NCBI	1	0	no_IT
Solanales	Solanaceae	10.1073/pnas	1	0	never_IT
Brassicales	Brassicaceae	10.1038/ng.2	0	0	never_IT
Brassicales	Brassicaceae	10.1038/ng.2	0	0	never_IT
Fagales	Betulaceae	NCBI	0	0	no_IT
Cyperales	Cyperaceae	NCBI	0	0	never_IT
Brassicales	Caricaceae	10.1038/natu	1	0	never_IT
Fabales	Fabaceae	10.1126/scier	1	0	no_IT
Fagales	Casuarinaceae	10.1126/scier	1	1	IT
Fagales	Fagaceae	NCBI	1	0	no_IT
Oxalidales	Cephalotaceae	10.1038/s415	0	0	never_IT
Fabales	Caesalpiniaceae	10.1126/scier	1	0	no_IT
Dicranales	Dicranaceae	Unpublished -	0	0	never_IT
Charales	Characeae	10.1016/j.cel	0	0	never_IT
Fabales	Caesalpiniaceae	10.1126/scier	1	1	no_IT
Caryophyllales	Chenopodiaceae	10.1038/natu	0	0	never_IT
Chlorokybales	Chlorokybaceae	10.1038/s414	0	0	never_IT
Chlamydomonales	Chlamydomonaceae	10.1126/scier	0	0	never_IT
Chlorellales	Chlorellaceae	10.1105/tpc.	0	0	never_IT

Gigartinales	Gigartinaceae	10.1073/pnas	0	0	never_IT
Sphaeropleales	Chromochloridaceae	10.1073/pnas	0	0	never_IT
Fabales	Fabaceae	10.1038/srep	1	1	IT
Sapindales	Rutaceae	10.1038/nbt.	1	0	never_IT
Cucurbitales	Cucurbitaceae	10.1038/ng.2	1	0	no_IT
Sapindales	Rutaceae	10.1038/nbt.	1	0	never_IT
Cucurbitales	Cucurbitaceae	NCBI	1	0	no_IT
Cucurbitales	Cucurbitaceae	10.1016/j.mc	1	0	no_IT
Cucurbitales	Cucurbitaceae	10.1073/pnas	1	0	no_IT
Cucurbitales	Cucurbitaceae	10.1016/j.mc	1	0	no_IT
Cucurbitales	Cucurbitaceae	10.1111/pbi.	1	0	no_IT
Cucurbitales	Cucurbitaceae	10.1038/ng.2	1	0	no_IT
Solanales	Convolvulaceae	NCBI	0	0	never_IT
Glaucocystales	Glaucocystaceae	10.1126/scier	0	0	never_IT
Cycadales	Cycadaceae	gymnoplaza	1	0	never_IT
Cucurbitales	Dasticaceae	10.1126/scier	1	1	no_IT
Apiales	Apiaceae	10.1038/ng.3	1	0	never_IT
Asparagales	Orchidaceae	10.1038/natu	0	0	never_IT
Caryophyllales	Caryophyllaceae	10.1093/dnar	0	0	never_IT
Rosales	Rhamnaceae	10.1126/scier	1	1	no_IT
Rosales	Rosaceae	10.1126/scier	1	1	no_IT
Chlamydomonales	Dunaliellaceae	10.1128/genc	0	0	never_IT
Nymphaeales	Nymphaeaceae	NCBI	0	0	never_IT
Brassicales	Brassicaceae	10.3389/fpls.	0	0	never_IT
Fabales	Fabaceae	10.1093/gigas	1	1	IT
Rosales	Moraceae	NCBI	1	0	no_IT
Rosales	Rosaceae	www.rosacea	1	0	no_IT
Lamiales	Oleaceae	10.1038/natu	1	0	never_IT
Rosales	Rosaceae	NCBI	1	0	no_IT
Rosales	Rosaceae	10.1093/gigas	1	0	no_IT
Cyanidiales	Galdieriaceae	10.1126/scier	0	0	never_IT
Ginkgoales	Ginkgoaceae	10.5524/100	1	0	never_IT
Fabales	Fabaceae	10.1038/natu	1	1	IT
Fabales	Fabaceae	NCBI	1	1	IT
Gnetales	Gnetaceae	10.5061/drya	0	0	never_IT
Malvales	Malvaceae	10.1038/natu	1	0	never_IT
Asterales	Asteraceae	10.1038/natu	1	0	never_IT
Malpighiales	Euphorbiaceae	NCBI	1	0	never_IT
Poales	Poaceae	10.1038/natu	1	0	never_IT
Rosales	Cannabaceae	10.1093/pcp/	1	0	no_IT
Malpighiales	Euphorbiaceae	NCBI	1	0	never_IT
Fagales	Juglandaceae	10.1111/tpj.1	1	0	no_IT
Klebsormidiales	Klebsomidiaceae	10.1038/ncor	0	0	never_IT
Fabales	Fabaceae	10.1093/gigas	1	1	IT
Cucurbitales	Cucurbitaceae	10.1111/tpj.1	1	0	no_IT
Malpighiales	Linaceae	NCBI	1	0	never_IT
Fabales	Fabaceae	10.1101/202	1	1	IT
Fabales	Fabaceae	10.1038/s414	0	1	no_IT
Fabales	Fabaceae	10.1111/pbi.	0	1	no_IT
Rosales	Rosaceae	NCBI	1	0	no_IT
Rosales	Rosaceae	www.rosacea	1	0	no_IT
Malpighiales	Euphorbiaceae	10.1038/nbt.	1	0	never_IT

Marchantiales	Marchantiaceae	10.1038/s415	1	0	never_IT
Marchantiales	Marchantiaceae	10.1038/s414	1	0	never_IT
Fabales	Fabaceae	10.1038/s414	1	1	IT
Zygnemales	Zygnematophyceae	10.1016/j.cel	0	0	never_IT
Mesostigmatales	Mesostigmataceae	10.1038/s414	0	0	never_IT
Mamiellales	Mamiellaceae	10.1126/scier	0	0	never_IT
Fabales	Fabaceae	10.1126/scier	1	1	IT
Cucurbitales	Cucurbitaceae	NCBI	1	0	no_IT
Sphaeropleales	Selenastraceae	10.1186/147	0	0	never_IT
Rosales	Moraceae	10.1038/ncor	1	0	no_IT
Fabales	Fabaceae	NCBI	1	1	IT
Zingiberales	Zingiberaceae	10.1093/data	1	0	never_IT
Proteales	Nelumbonaceae	10.1186/gb-2	0	0	never_IT
Solanales	Solanaceae	10.1094/MPN	1	0	never_IT
Fabales	Fabaceae	10.1126/scier	1	0	no_IT
Nymphaeales	Nymphaeaceae	NCBI	0	0	never_IT
Poales	Poaceae	10.1093/nar/	1	0	never_IT
Mamiellales	Mamiellaceae	10.1073/pnas	0	0	never_IT
Mamiellales	Mamiellaceae	10.1186/147	0	0	never_IT
Rosales	Cannabaceae	10.1073/pnas	1	1	no_IT
Desmidiales	Peniaceae	10.1016/j.cel	0	0	never_IT
Solanales	Solanaceae	10.1038/npla	1	0	never_IT
Asparagales	Orchidaceae	10.1038/natu	0	0	never_IT
Fabales	Fabaceae	10.1038/ng.3	1	1	IT
Funariales	Funariaceae	10.1111/tpj.1	0	0	never_IT
Pinales	Pinaceae	10.1038/natu	0	0	never_IT
Pinales	Pinaceae	10.1093/bioir	0	0	never_IT
Pinales	Pinaceae	gymnoplaza	0	0	never_IT
Pinales	Pinaceae	gymnoplaza	0	0	never_IT
Pinales	Pinaceae	gymnoplaza	0	0	never_IT
Pinales	Pinaceae	10.1534/gene	0	0	never_IT
Fabales	Fabaceae	10.1038/s415	1	1	IT
Malpighiales	Salicaceae	NCBI	1	0	never_IT
Malpighiales	Salicaceae	NCBI	1	0	never_IT
Malpighiales	Salicaceae	10.1126/scier	1	0	never_IT
Porphyridiales	Porphyridiaceae	10.1038/ncor	0	0	never_IT
Bangiales	Bangiaceae	10.1073/pnas	0	0	never_IT
Rosales	Rosaceae	10.1093/gigas	1	0	no_IT
Fabales	Fabaceae	NCBI	1	1	IT
Rosales	Rosaceae	10.1093/dnar	1	0	no_IT
Rosales	Rosaceae	10.1111/tpj.1	1	0	no_IT
Rosales	Rosaceae	NCBI	1	0	no_IT
Rosales	Rosaceae	10.1038/ng.2	1	0	no_IT
Rosales	Rosaceae	NCBI	1	0	no_IT
Pinales	Pinaceae	10.1534/g3.1	0	0	never_IT
Rosales	Rosaceae	NCBI	1	0	no_IT
Rosales	Rosaceae	NCBI	1	0	no_IT
Rosales	Rosaceae	10.1371/jour	1	0	no_IT
Bangiales	Bangiaceae	10.1371/jour	0	0	never_IT
Fagales	Fagaceae	NCBI	1	0	no_IT
Fagales	Fagaceae	10.1111/175	1	0	no_IT
Rosales	Rhamnaceae	NCBI	1	0	no_IT

Ericales	Ericaceae	10.1038/s414	0	0	never_IT
Ericales	Ericaceae	10.1093/gbe/	0	0	never_IT
Malpighiales	Euphorbiaceae	10.1038/nbt.	1	0	never_IT
Rosales	Rosaceae	10.1038/s415	1	0	no_IT
Rosales	Rosaceae	10.1111/tpj.1	1	0	no_IT
Salviniales	Salviniaceae	10.1038/s414	0	0	never_IT
Sapindales	Anacardiaceae	10.1093/gigas	1	0	never_IT
Selaginellales	Selaginellaceae	10.1038/s414	1	0	never_IT
Selaginellales	Selaginellaceae	10.1126/scien	1	0	never_IT
Poales	Poaceae	10.1038/nbt.	1	0	never_IT
Solanales	Solanaceae	10.1038/natu	1	0	never_IT
Solanales	Solanaceae	10.1038/ng.3	1	0	never_IT
Poales	Poaceae	10.1111/tpj.1	1	0	never_IT
Fabales	Fabaceae	NCBI	1	1	IT
Sphagnales	Sphagnaceae	Phytozome	0	0	never_IT
Zygnemales	Zygnematophyceae	10.1016/j.cel	0	0	never_IT
Caryophyllales	Amaranthaceae	bvseq.molgen	0	0	never_IT
Alismatales	Araceae	10.1038/ncon	0	0	never_IT
Brassicales	Cleomaceae	10.1105/tpc.1	0	0	never_IT
Malvales	Malvaceae	10.1186/gb-2	1	0	never_IT
Rosales	Cannabaceae	10.1073/pnas	1	0	no_IT
Poales	Poaceae	10.1093/gigas	1	0	never_IT
Fabales	Fabaceae	10.1038/srep	1	1	IT
Fabales	Fabaceae	NCBI	1	1	IT
Lamiales	Lentibulariaceae	10.1073/pnas	0	0	never_IT
Lamiales	Lentibulariaceae	10.3390/ijms	0	0	never_IT
Fabales	Fabaceae	10.1038/srep	1	1	IT
Fabales	Fabaceae	10.1038/ncon	1	1	IT
Fabales	Fabaceae	10.1093/gigas	1	1	IT
Fabales	Fabaceae	10.1111/tpj.1	1	1	IT
Chlamydomonales	Volvocaceae	10.1126/scien	0	0	never_IT
Poales	Poaceae	10.1105/tpc.1	1	0	never_IT
Rosales	Rhamnaceae	10.1038/ncon	1	0	no_IT
Alismatales	Zosteraceae	10.1038/natu	0	0	never_IT
