# Supplementary material for: Stabilization of membrane topologies by proteinaceous remorin scaffolds
Source: Nat Commun. 2023 Jan 19;14:323. doi: 10.1038/s41467-023-35976-5 (PMC9852587; doi:10.1038/s41467-023-35976-5)
Supplement: Supplementary file 7 — Supplementary Data 4 [file 41467_2023_35976_MOESM7_ESM.pdf]

Supplementary Table 4: Primers used in the study.

**For qPCR**	
MtREM2.1-F	CCTGTTGTGGAAAAGGAATCTG
MtREM2.1-R	TATTATCAGCATGATCATCTG
gPCR_Ubiquitin_1F	GCAGATAGACACGCTGGGA
gPCR_Ubiquitin_1R	AACTCTTGGGCAGGCAATAA
**MtREM2.1 Promoter**	
MtREM2.1-F	TTGAAGACTTTACGGGTCTCAGCGGATTTATTAAATGAAATGGGCGATCC
MtREM2.1-R	ATGAAGACTTCAGAGGTCTCACAGAGACTTGTTCTGTGGATATATACAC
**MtREM2.1 coding sequence**	
MtREM2.1-F	TTGGTCTCACACCGGAGAATCAGAAGGTTCCAGC
MtREM2.1-R	TTGGTCTCACCTTAGCACTAAAGCATCCAAAC
**AtREM2.1 coding sequence**	
AtREM2.1-F	ATGGTCTCACACCATGCATATGGGACAAAATAAACC
AtREM2.1-R	TAGGTCTCTGATTTTAGAAACATCCACAAGTTTTGG
**Protein purification**	
His-SYMREM1(pDEST17)-F	ATGGTCTCACACCATGGAAGAATCGAAAAACAAAC
SYMREM1-(pDEST17)R	TAGGTCTCTCCTTCTAACTGAAAAACCTTAAACCGC
His-GFP-SYMREM1(pDEST17)-F	ATGGTCTCACACCATGGTGAGCAAGGGCGAGGAGC
SYMREM1(pET303)-F	AATTTTGTTTAACTTTAAGAAGGAGGTATGGAAGAATCGAAAAACAAAC
SYMREM1(pET303)-R	AGCTCTTCACCTTTACTGACCATTCTAGaACTGAAAAACCTTAAACCGCT
SYMREM1^{IDR}(pET303)-F	AATTTTGTTTAACTTTAAGAAGGAGGTATGGAAGAATCGAAAAACAAAC
SYMREM1^{IDR}(pET303)-R	CAGCTCTTCACCTTTACTGACCATTCTAGaTTCAACCCTTGCTAGTAC
SYMREM1^{Cterm}(pET303)-F	AATTTTGTTTAACTTTAAGAAGGAGGTatgTCGCAGAAAAGATTGGCTTTAAT
SYMREM1^{Cterm}(pET303)-R	AGCTCTTCACCTTTACTGACCATTCTAGaACTGAAAAACCTTAAACCGCT
